# Controlling feeding behavior by chemical or gene-directed targeting in the brain: what's so spatial about our methods?

**DOI:** 10.3389/fnins.2013.00182

**Published:** 2013-12-18

**Authors:** Arshad M. Khan

**Affiliations:** ^1^UTEP Systems Neuroscience Laboratory, Department of Biological Sciences, Border Biomedical Research Center, University of Texas at El PasoEl Paso, TX, USA; ^2^Neurobiology Section, Department of Biological Sciences, University of Southern CaliforniaLos Angeles, CA, USA

**Keywords:** injections, optogenetic, pharmacogenetic, pharmacosynthetic, mapping, stereotaxic, neuroinformatics, DREADD

## Abstract

Intracranial chemical injection (ICI) methods have been used to identify the locations in the brain where feeding behavior can be controlled acutely. Scientists conducting ICI studies often document their injection site locations, thereby leaving kernels of valuable location data for others to use to further characterize feeding control circuits. Unfortunately, this rich dataset has not yet been formally contextualized with other published neuroanatomical data. In particular, axonal tracing studies have delineated several neural circuits originating in the same areas where ICI injection feeding-control sites have been documented, but it remains unclear whether these circuits participate in feeding control. Comparing injection sites with other types of location data would require careful anatomical registration between the datasets. Here, a conceptual framework is presented for how such anatomical registration efforts can be performed. For example, by using a simple atlas alignment tool, a hypothalamic locus sensitive to the orexigenic effects of neuropeptide Y (NPY) can be aligned accurately with the locations of neurons labeled by anterograde tracers or those known to express NPY receptors or feeding-related peptides. This approach can also be applied to those intracranial “gene-directed” injection (IGI) methods (e.g., site-specific recombinase methods, RNA expression or interference, optogenetics, and pharmacosynthetics) that involve viral injections to targeted neuronal populations. Spatial alignment efforts can be accelerated if location data from ICI/IGI methods are mapped to stereotaxic brain atlases to allow powerful neuroinformatics tools to overlay different types of data in the same reference space. Atlas-based mapping will be critical for community-based sharing of location data for feeding control circuits, and will accelerate our understanding of structure-function relationships in the brain for mammalian models of obesity and metabolic disorders.

## 1. Introduction

In the past two decades, our abilities to acutely manipulate neural substrates controlling behavior in the living animal have transformed substantially. New transgenic methods to control brain circuits, such as *in vivo* site-specific recombinase technology (Lakso et al., 1992; Orban et al., 1992; Gu et al., 1994), viral-mediated expression or RNA interference (RNAi) (Davidson et al., 1993; Chamberlin et al., 1998; Hommel et al., 2003), optogenetics (Boyden et al., 2005; Lima and Miesenböck, 2005; Bernstein et al., 2012) and pharmacosynthetics (Armbruster et al., 2007; Farrell and Roth, 2013), are now being used to address how complex, goal-directed behaviors occur from organized networks of neurons. At present, only a few laboratories have used these methods to study feeding behavior. These state-of-the-art methods are now rapidly finding their place in our scientific literature alongside studies involving acute chemical microinjection methods to control behavior that were first developed over a century ago, and first used for studying acute feeding control in 1954.

In this review, these methods will be examined in relation to the neuroanatomical information they have provided to scientists trying to identify brain regions and circuits controlling feeding behavior. Section 2 includes a brief history of central microinjection methods, focusing on injection sites and maps of feeding control regions. I found the excellent handbook by Myers (1974) to be an indispensible resource for most studies published between 1915 and 1972. In Section 3, I review gene-directed studies used to study feeding behavior, including site-specific recombinase technology, viral-mediated expression, and RNAi, optogenetics, and pharmacosynthetics; highlighting special experimental considerations related to these techniques for documenting injection and probe sites, as well as populations of neurons activated or transduced by viruses. In Section 4, a framework is proposed for examining location data in the brain in a manner that allows one to interrelate such data from multiple studies of the same brain region. I compare location data obtained from chemical injection studies with those using newer gene-directed methods. In Section 5, I argue that within studies using intracranial injection methods, the careful documentation of the locations of the injection sites or the cell populations affected by such injections is critical for interrelating these location data with data found in other types of studies. For example, neuroanatomical tract-tracing studies have provided much information concerning the circuit connections of neurons in key feeding control regions of the brain. Many of these regions have also been targets of various intracranial central injection methods. Placing the injection sites found in one study in anatomical registration with traced circuits documented in another can aid in the formulation of plausible, constrained hypotheses concerning how functional feeding circuits are controlled and organized. The proposal is made, also in Section 5, that neuroinformatics approaches provide a set of powerful tools to analyze diverse sets of data that are linked by the location information they each contain.

In this article, the focus is not on the circuits controlling food intake (i.e., the biology), but rather how to contextualize neuroanatomical data with chemical and/or gene-directed microinjection data (i.e., methodology). Several cogent reviews cover details about feeding (and related) control circuits (Stevenson, 1969; Myers, 1974; Swanson, 1987, 2000c; Loewy and Spyer, 1990; Blessing, 1997; Elmquist et al., 1999; Watts and Swanson, 2002; Llewellyn-Smith and Verberne, 2011). Since most chemical injection studies concerning feeding control have been focused on manipulations of rat hypothalamus, this structure is emphasized in this article, but a few key studies performed in other brain regions [e.g., prefrontal cortex, nucleus accumbens, ventral tegmental area (VTA), nucleus of the solitary tract] and in other species (goat, rabbit, guinea pig, mouse) are also highlighted. For newer methods involving viral injections, the reader will find attention shifted more to the mouse, reflecting the greater importance this animal model now has for scientists investigating feeding control. Before we discuss these topics, it is useful to compare chemical injection studies with newer gene-directed approaches used to control behavior.

### 1.1. Intracranial injection methods to acutely control behavior

For our purposes here, the terms intracranial chemical injections (ICI) and intracranial gene-directed injections (IGI) will be used to differentiate traditional, acute chemical injection methods (Myers, 1974) from recently developed methods involving cell type-specific insertion of engineered molecules, such as *in vivo* site-specific recombinase technology (Lakso et al., 1992; Orban et al., 1992; Gu et al., 1994), viral-mediated RNA expression or interference (Davidson et al., 1993; Chamberlin et al., 1998; Hommel et al., 2003), optogenetics (Boyden et al., 2005; Lima and Miesenböck, 2005; Bernstein et al., 2012), and pharmacosynthetics (Armbruster et al., 2007; Farrell and Roth, 2013). While not fully inclusive of the possible combinatorial strategies that can be employed to control engineered molecules in the brain, the term IGI is being used deliberately to emphasize those variants of gene-directed methods that specifically involve *injections* of various constructs into the brain. Also, *in vivo* microdialysis and other related methods (push-pull perfusion, amperometry, etc.) are not covered in this article, but the concepts discussed here also apply to these methods.

Although ICI and IGI methods were developed nearly a century apart from one another (Maxwell, 1906a,b; Hommel et al., 2003; Balthasar et al., 2005; Adamantidis et al., 2007; Alexander et al., 2009), both rely on the use of intracranial injections to either deliver chemical ligands (ICI) or various genetically engineered sequences (IGI), such as those encoding Cre recombinase, short hairpin RNA (shRNA), neuropeptides, enzymes, ion channels, ion transporters, or receptors; deep within brain tissue to control behavioral output. With ICI, the chemicals travel to their targets either from crystalline or wax deposits slowly dissolving in the brain after implantation, or as pre-dissolved solutes within inert “vehicle,” or carrier solutions; in rare instances, such chemicals are also delivered by being secreted from tissue grafts implanted at the site of interest (e.g., Flerkó and Szentágothai, 1957; see Myers, 1974). With IGI, the genetic sequences are packaged within modified viruses that are injected into the brain (e.g., Davidson et al., 1993), where the sequences are transduced and expressed within neuronal populations, often in a promoter-specific fashion (e.g., Zhang et al., 2010). Once expressed, these molecules can be engaged in various ways that depend upon the specific IGI approach used. For site-specific recombinase technology [(Sauer, 1987; Sauer and Henderson, 1988; Lakso et al., 1992; Orban et al., 1992; Gu et al., 1994); reviewed in Sauer (1998)], the viruses deliver a sequence encoding the enzyme, Cre recombinase, to cells in a transgenic animal harboring LoxP sequences genetically inserted to flank genes intended for deletion, insertion, translocation, or inversion. For site-specific gene deletion, the expressed Cre recombinase initiates splicing of the sequence at LoxP sites within the specific cells where it has been delivered by the virus. In viral-mediated RNAi, shRNA inhibits the expression of particular molecules within the cell (Hommel et al., 2003). In optogenetic methods, neurons can be activated or inhibited via light pulses of specific wavelengths delivered from implanted optical fibers situated in the vicinity of the expressed light-sensitive ion channels (Boyden et al., 2005). In pharmacosynthetic methods (Armbruster et al., 2007; Farrell and Roth, 2013), activation or inhibition of “designer” receptors inserted into cells by viral transduction occurs by “designer” ligands injected centrally or that are delivered systemically and cross the blood-brain barrier, such as the prototypical synthetic molecule, clozapine-*N*-oxide (CNO).

Another similarity between ICI and IGI methods is that they often involve combinations of intracranial manipulations. Thus, prior to the injection of chemicals or viruses, these techniques may first require the implantation of chronically indwelling guide cannulas that facilitate rapid and reproducible insertion of the injector needles or optical fibers into brain tissue. The first tissue perturbation would therefore be from the cannula implantation, and the second from the injector needle or fiber itself. In such cases, the tissue is subjected to more than one invasive manipulation. As discussed in the next section, the tissue itself supplies a critical type of datum in ICI and IGI studies: location information about the experimental manipulation.

### 1.2. Sites and cells: the only visual references left by ICI and IGI about location

*“… [N]etwork visualization is also the cartography of the indiscernible, depicting intangible structures that are invisible and undetected by the human eye, from eccentric visualizations of the World Wide Web to representations of the brain*'*s neural network. In some cases, the maps of these hidden structures are the only visual reference we have, constituting its own alternative reality.”*

*— Manuel Lima (2011)*

*Visual Complexity: Mapping Patterns of Information*

ICI and IGI studies of feeding behavior are conducted to achieve certain goals. First, there is a need to understand the qualitative and quantitative aspects of feeding behavior itself, such as feeding duration, latency to eat, timing and sizes of meals, inter-meal interval durations, or responses to conditioned cues (Brobeck, 1955; Le Magnen, 1985). Second, there is also a need to localize the effects of intracranial manipulations to a set of defined neural substrates. This is done by “identifying” brain regions and the circuits within them that are responsible for producing the behavior observed, based on probe locations in the brain.

It is this process of “identification” that is the main concern of this review. What can be identified within the tissue after using ICI or IGI methods to mark the site of experimental stimulation or suppression of feeding behavior? As will be detailed in Section 4.4.9.1. ICI and IGI methods leave several physical signs of experimental perturbation: (1) tissue displacement from the delivery of solutions carrying viral particles or dissolved chemicals; (2) tissue damage from glass micropipettes, needles, or optical fibers; (3) tissue damage from any indwelling cannulas used to guide probes to target locations; (4) histochemically detectable reporter molecules or tracers expressed or visualized in association with experimental manipulation or viral transduction.

Echoing Lima's statement above, the only visual references that usually remain about the location of an ICI or IGI manipulation, from which we can try and begin inferring the locations of underlying feeding circuits, is what has been documented within the published study for others to utilize. Importantly, it is useful to consider this published documentation as the type of “alternative reality” Lima describes. By being consciously aware of the rather underappreciated notion that the visual documentations of injection sites and cell location data—within a published report—constitute a proxy for that which is unseen and inaccessible in the brain, we might be more attentive in making sure that our published descriptions of such data, as a community, are as informative as possible. In doing so, we are ensuring that future scientists can fully access our datasets and bring them into register with their own as yet uncollected data. I approach the ingestive behavior community here with this argument, but it also has been made elsewhere for functional human brain imaging data (Devlin and Poldrack, 2007; Toga and Thompson, 2007; Van Essen and Dierker, 2007). In Sections 4 and 5, we will see how visual representations of data from the same brain region can be interrelated across published studies. Before beginning this topic, let us examine visual representations historically used to document intracranial microinjections (ICI methods) controlling feeding behavior.

## 2. The experimental control of food intake using ICI: a brief history

From the 1900s to the 1950s, various electrical stimulation or lesioning methods were used to investigate the neural substrates underlying feeding, autonomic and neuroendocrine control [e.g., the work of Karplus and Kreidl (*Gehirn und Sympathicus*, Parts I–VIII: 1909, 1910, 1911, 1918, 1924, 1927, 1928; Einthoven et al., 1927); the work of Hess in the 1920s and 1930s, reviewed by Hess (1954, 1956, 1957), Camus and Roussy, 1920; Ranson et al., 1935; Hetherington and Ranson, 1939, 1940; Anand and Brobeck, 1951a,b; Delgado and Anand, 1953; reviewed by Ingram (1939); Stevenson (1969)]. Moreover, studies of self-stimulation since the 1950s [(Olds and Milner, 1954; Hoebel and Teitelbaum, 1962); reviewed by Olds (1977); Wise (2005); Berthoud and Münzberg (2011)], linked feeding control to larger ideas about drive and reinforcement. Alongside these methods, ICI methods also contributed richly to our understanding of how neural substrates are causally linked to complex, goal-directed behaviors such as feeding. A few pioneering scientists first established this causal link during the 1950s and 1960s, by acutely delivering chemical substances into the brain of the freely moving animal to control feeding behavior (Larsson, 1954; Grossman, 1960; Epstein, 1960a,b). In this section, I trace the origins of such chemical control and note a few key historical studies that will be discussed within a larger theoretical framework in Section 4 and 5.

### 2.1. Nineteenth century antecedents

The chemical control of brain function originated within the larger intellectual discourse of the early to mid-nineteenth century concerning the localization of nervous system function (Fearing, 1930; Young, 1990). Table 1 shows some key developments during this time for chemical control studies of the brain. Intracranial injections were ostensibly popularized by these developments, which laid the groundwork for a more careful examination of chemical agents upon central nervous system function. By the early twentieth century, chemical studies became practical laboratory textbook exercises (e.g., Jackson, 1917; Sherrington, 1919; Barbour, 1932).

**Table 1:** **Early studies investigating the chemical control of the brain**.

**Study**	**Summary of findings**
Flourens, 1832	Topically applied *l'huile essentielle de térébenthine* and *teinture de Rousseau* (turpentine oil and opium, respectively) on rabbit cerebral lobes and observed that these two substances, applied to the same region of the brain, trigger different behavioral outcomes, locomotion and torpor.
Sechenov, 1863	Demonstrated that chemically stimulating frog sciatic nerve with liquid or crystalline salt could affect excitability of the medulla. See also Roitbak (1980).
Landois, 1887; Bickel, 1898	Applied powdered creatine, creatinine, urea derivatives, or other compounds to the surface of the cerebral hemispheres and produced contralateral contortions of the body.
Roux and Borrel, 1898	Introduced a neutralizing tetanus antitoxin into two compartments of a tetanus-infected guinea pig: the peripheral circulation and the brain. Building upon a study by Wassermann and Takaki (1898), who provided evidence that tetanus toxin binds to nervous tissue, the scientists showed that introducing the antitoxin into the brain conferred immunity and lowered mortality in the animals to a greater extent than peripheral administration of the antitoxin.
Chauffard and Quénu, 1898	On April 26, 1898, performed the first intracerebral injections of tetanus antitoxin via hypodermic needles on human patients. See also Semple (1899). Similar treatments on human patients were made by a number of clinicians, but to varying degrees of success. As Wilson (1997) describes, the practice was soon abandoned after it was reported that intravenous or subcutaneous injections were actually superior means of antitoxin delivery.
Aronsohn and Sachs, 1885	Demonstrated that a needle puncture of the striatum could produce a temperature increase in the animal.
Meltzer, 1900	Among the first to perform intracerebral injections in animals, but did not document injection site locations. For related comments, see Ehrlich (1899); Coriat (1905).
Donath, 1904	Among the first to perform intracerebral injections in animals, but did not document injection site locations.
Maxwell, 1906a,b	Used glass capillary tubes to deliver creatine, sodium citrate and other solutions into the gray and white matter of rabbits.
Barbour, 1912	Introduced an ingenious closed cannula system for routing cool water through the brain (but not into it) in order to alter its temperature (which it did).
Hashimoto, 1915	Used an open guide cannula system for intracranial injection, perhaps for the first time, when blood serum was delivered into the brain of rabbits to examine the ensuing febrile response.
Sahlgren, 1934	Demonstrated sleep-producing effects after intracranial injections of sodium phenobarbital (*Luminalnatrium*) in rabbits.
Masserman, 1937	Began reporting autonomic responses in cats following injections of various chemical substances into the brain, some with known actions as neurotransmitters (Masserman, 1937, 1939a,b; Masserman and Haertig, 1938a,b; Masserman and Jacobson, 1940; Masserman et al., 1940). Although these studies confirmed the hypothalamic control of autonomic function reported during electrical stimulation (e.g., Karplus and Kreidl, 1909, 1910, 1911; Ranson et al., 1935; also see Hess, 1954, 1956, 1957), they did not include documentation of the injection sites, and included only brief notes concerning the nomenclature and stereotaxic atlas used (Clarke and Henderson, 1911), or in some instances (e.g., Masserman and Haertig, 1938a) the hypothalamic regions examined.

*This is a list of selected studies investigating the chemical control of the brain in the nineteenth and early twentieth centuries*.

### 2.2. A century of injection sites

**Figure 1:**
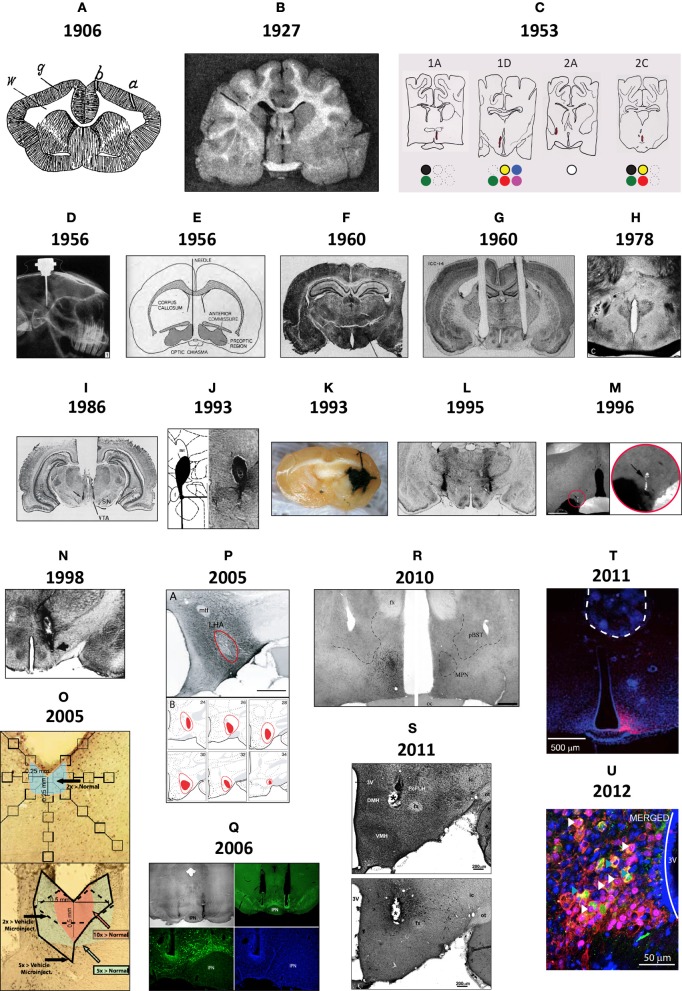
**(A)** This drawing is of a frontal section from a rabbit, first published in 1906 by Samuel S. Maxwell at the University of California (Maxwell, 1906b). It shows injection sites made into the cerebral cortex, either at the superficial level of the upper gray (*g*) matter (needle track shown by *a*), or more deeper into the white (*w*) matter (needle track shown by *b*). Injections of calcium oxalate were made to evoke movements in the animal; these were only observed with injections into the white matter. Maxwell also performed similar experiments on cats. **(B)** This drawing is Figure 1 of Demole (1927), which provides a view of how injection sites were marked. CaCl_2_ injections were made into the anterior hypothalamus of cats to produce sleep. Note the needle track and its final termination, the injection site, are marked by black dye that was included in the CaCl_2_ solution at the time of injection. Formalin-fixed tissue was used so that the tissue was relatively clear of blood, and gross anatomical landmarks were used within the tissue to aid in documenting the location. **(C)** A portion of the hand drawings included in the unpublished medical school thesis of Eglin (1953), reproduced here with permission. Needle tracks are colored red in each drawing. The colored dots have been added by the present author to denote the observations Eglin recorded for each of the corresponding injections: white circles: no effect; black: pupillary dilation; green: locomotion; yellow: piloerection; red: growling; blue: licking; magenta: salivation. **(D)** X-ray image of guide cannula and injector assembly within a rabbit test subject's skull; the acrylic holding the assembly has been drawn on the image as a white outline (von Euler and Holmgren, 1956). **(E)** A diagram showing the representative location of Fisher's injection placements (1956) in the medial preoptic region to trigger switching between sexual and maternal behaviors in male rats (the diagram is from a review by Fisher published in 1964). **(F)** The Nissl-counterstained photomicrograph included in Grossman's report (1960) of norepinephrine feeding following crystalline administration of norepinephrine or carbachol into the perifornical hypothalamus. **(G)** The Nissl-counterstained photograph showing needle tracks from the studies of Epstein (1960b). **(H)** A modification of the Klüver–Barrera stain was used by Leibowitz (1975) to stain the tissue from subjects given amphetamine injections in to the lateral hypothalamus. This photo is from Figure 2C of her study. **(I)** Representative injection site in the vicinity of the ventral tegmental area, from Figure 4C of Cador et al. (1986), where enkephalin injection stimulated food intake. **(J)** Injection site and accompanying map (from Paxinos and Watson, 1986) where 10 nl of NPY was injected into the perifornical hypothalamus to stimulate food intake (from Stanley et al., 1993a,b,c). **(K)** Beta-galactosidase-stained needle track and injection site marking extent of viral induction following injection of an adeno-associated virus by Davidson et al. (1993) within an unstained brain. **(L)** Bilateral injection sites where the GABA_A_ receptor agonist, muscimol, was injected into the lateral hypothalamus to attenuate feeding produced by subsequent injections of glutamate receptor antagonists into the nucleus accumbens within the same animal (Maldonado-Irizarry et al., 1995). **(M)** Injection site produced by Scammell et al. (1996) within the preoptic region where prostaglandins induce a febrile response; the injection site is marked by fluorescent beads (enlarged by the present author in the inset to the right). **(N)** Injection site in the perifornical hypothalamus where cyclic AMP analogs stimulate feeding (Gillard et al., 1998). **(O)** Example of a vehicle-injected (top) and DAMGO-injected (bottom) subject stained for Fos; Fos “plume” areas, as measured by the authors, are indicated diagrammatically (from Peciña and Berridge, 2005). **(P)** Fluorogold injection site and accompanying maps in Swanson atlas space (Swanson, 1999) reported by Petrovich et al. (2005), which retrogradely labeled neurons subsequently examined for activation responses during cue-initiated feeding. **(Q)** Injection site characterization within the ventral tegmental area by Hommel et al. (2006; from their supplementary data) using immunocytochemistry staining patterns for tyrosine hydroxylase (green panels) and the nuclear stain, DAPI (blue). **(R)** Injection sites marked by immunostaining for the androgen receptor following delivery of either vehicle (right side) or testosterone (left side); from Williamson et al. (2010) **(S)** Photomicrographs reproduced from Lin et al. (2011) in which a Nissl stain has been applied to a tissue section to help localize an injection site made into the medial hypothalamus (*S, top*), along with an adjacent tissue section stained for the peptide hypocretin/orexin (*S, bottom*). **(T)** Location of the optical fiber used to drive Channelrhodopsin-2 (ChR2)-expressing AgRP neurons in the mouse arcuate hypothalamus and stimulate feeding behavior. This fluorescence photomicrograph shows the ChR2-expressing neurons in red, as visualized by the reporter molecule, mCherry. The tissue has been counterstained blue using the nuclear label, DAPI. From the supplementary data in Aponte et al. (2011). **(U)** Fos activation (magenta) in POMC neurons (green) transduced with Channelrhodopsin-2 (tdtomato reporter expression; red) in the mouse ARH (from supplementary data in Atasoy et al., 2012). Figures are reproduced with permission of their original publishers, as noted here: Panel **(D)** (John Wiley & Sons, Ltd.); **(E)** (Scientific American); **(F)** (The American Association for the Advancement of Science); **(G)** (The American Physiological Society); **(H,I** and **S)** (Elsevier); **(J,L–P**, and **R)** (Society for Neuroscience); **(K,T** and **U)** (Nature Publishing Group); and **(Q)** (Cell Press, Inc.).

Figure 1 shows brain injection sites chronologically appearing in selected scientific studies from the early 1900s to the present day, beginning with Maxwell's documentation of needle tracks in the brain (1906a,b, Table 1), perhaps the first to be published (Figure 1A). In the early twentieth century, several scientists working in European laboratories were focused on ascertaining the locations of “centers” for the production of heat (*Wärmzentrum*) and sleep (*Schlafzone*). For example, while trying to study how calcium chloride injections might produce a febrile response in cats, Demole (1927) found that the injections produced sleep and that potassium chloride injections triggered arousal. Injections were marked using carmine or black dye (Figure 1B).

Bengt Andersson in Sweden first demonstrated the intracranial chemical control of a motivated behavior by showing that drinking behavior could be elicited by sodium chloride injections into the goat diencephalon (Andersson, 1952, 1953; Andersson et al., 1958). In 1954, Andersson's student, Stig Larsson, reported that an intrahypothalamically injected sodium chloride solution also triggered feeding in sheep or goats. In the wake of these findings, two Yale University laboratories, directed by Neal Miller and Paul MacLean, respectively, began exploring central injection techniques (see Miller et al., 1955, 1956; Miller, 1956, 1957, 1965, 1992; MacLean, 1957a,b). In 1953, James Eglin, Jr., a MacLean lab student, provided in his completed medical school thesis what was perhaps the first anatomic documentation of injection sites producing autonomic responses by chemical injection. Unfortunately, Eglin's drawings (some reproduced for the first time in Figure 1C) were never published. von Euler and Holmgren (1956) took X-ray images of the implanted skull (Figure 1D) to show cannula locations for their thyroxine injections into the anterior pituitary, which inhibited radiolabeled iodine release from the thyroid gland. That same year, Fisher reported that intrahypothalamic testosterone induced nest building and “maternal care” behaviors in male rats; conversely, co-injected estrogen and progesterone induced heat in female rats (Fisher, 1956 also see Fisher, 1964; Figure 1E). Also, intrahypothalamically grafted ovary fragments (to introduce estrogen) suppressed rat gonadotropin secretion (Flerkó and Szentágothai, 1957).

In 1960, Sebastian Grossman, a Miller lab graduate student, reported that feeding or drinking could be elicited in rats receiving intrahypothalamic crystalline deposits of norepinephrine (NE) or carbachol, respectively. In parallel work, Alan N. Epstein at the University of Pennsylvania reported similar findings, albeit with procaine hydrochloride, hypertonic saline, and dextrose; substances without “neurotransmitter” actions (Epstein, 1960a,b). Both investigators photographed injection sites within Nissl-stained tissue (Figures 1F,G). Although many have used Nissl stains to help visualize injection site locations in brain tissue, a few have combined these with other treatments. For example, Leibowitz (1975) used a protocol (Wolf and Yen, 1968) modified from the original method of Klüver and Barrera (1953) to visualize anterior lateral hypothalamic sites where amphetamine suppresses feeding in rats, by labeling cells (using a Nissl stain) and myelin sheaths (using Luxol Fast Blue) in the same tissue (Figure 1H). Finally, extending work done by the Stanley laboratory establishing a role for glutamate and its receptor agonists in feeding control through actions within the lateral hypothalamic area (Stanley et al., 1993a,b,c, 1996; Khan et al., 1999, 2004; Duva et al., 2001, 2002, 2005; Hettes et al., 2003, 2007, 2010; see Stanley et al., 2011), Li et al. (2011) have carefully delineated the locations of feeding-sensitive sites for the glutamate receptor agonists NMDA and AMPA by using rigorous methods for situating the locations of their hypothalamic injections that triggered feeding behavior (Figure 1S).

#### 2.2.1. A note on peptides and feeding

The 1970s marked early efforts to deliver peptides, such as insulin, directly into the hypothalamus (Debons et al., 1970; Panksepp and Nance, 1972). Crystalline insulin deposited into the ventromedial hypothalamus reduced food intake in normal and diabetic rats (Hatfield et al., 1974). In contrast, β-endorphin stimulated feeding when injected into this region (Grandison and Guidotti, 1977). Documenting peptide injection sites gained currency in the 1980s. Cador et al. (1986), for example, found enkephalin injected into the VTA had a marked effect on feeding (Figure 1I). Similarly, following reports that intracerebroventricular neuropeptide Y (NPY) injection stimulated food intake (Clark et al., 1984; Levine and Morley, 1984), Stanley and Leibowitz (1984) reported feeding produced by NPY injected directly into the hypothalamic paraventricular nucleus (also see Stanley et al., 1986), and that the perifornical hypothalamus is the region most sensitive to NPY's effects on feeding (Stanley et al., 1993a,b,c) (Figure 1J). Several studies have since reported feeding induced or suppressed by peptides, including: bombesin, cholecystokinin, hypocretin/orexin (H/O), Agouti-related peptide (AgRP), melanin-concentrating hormone, alpha-melanocyte stimulating hormone, and ghrelin (reviewed by Kalra et al. (1999); Berthoud and Morrison (2008)].

### 2.3. A century of maps

For ICI methods, issues concerning the spread of injected solutions led to scientists using more sophisticated “cannula mapping” strategies to isolate the site(s) most sensitive to the autonomic or behavioral effects of injected solutions. Any injection site photographs included in these studies were now accompanied by location data “mapped” to standardized brain atlases.

#### 2.3.1. Early maps

**Figure 2:**
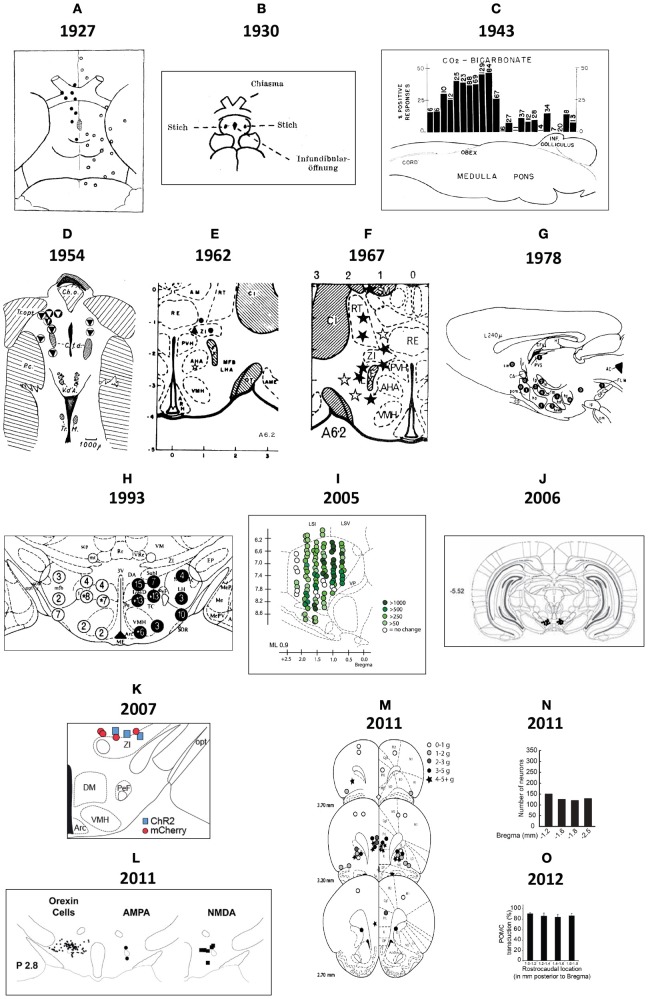
**(A)** This drawing is Figure 9 of Demole (1927), which provides what is likely the first published map of central chemical injection sites for the hypothalamus. CaCl_2_ injections were made into the anterior hypothalamus of cats to produce sleep. The ventral surface of the brain is shown, with locations of effective (*black circles*) and ineffective (*white circles*) injection sites. Note that the effective injection sites cluster near the optic chiasm and optic tracts, in the vicinity of the preoptic area and the anterior hypothalamus. **(B)** Drawing of injection sites near the infundibular region from the study by Clöetta and Fischer (1930). The labels are in the German language; injection sites are labeled “*Stich*.” **(C)** A generalized diagram by Comroe (1943) showing functional activation of respiration as a result of injections of bicarbonate solutions into various hindbrain locations. The responses are plotted in relation to the sagittal drawing below them to denote from where the responses were elicited. **(D)** This drawing is Figure 11 of Larsson (1954), which shows injection sites where sodium chloride administration was effective in triggering food intake (white circles with black triangles) or rumination (black circles with white triangles) in a goat. The injection sites are mapped with respect to “gross anatomic” visualizable space, as described in the text. Abbreviations: *C. a*., anterior commissure; *C. f. d*., columna fornix descendens; *Ch. Opt*., optic chiasm; *C. M*., mammillary body; *Hyp*, hypophysis with *pars post*. and *pars ant*. being the posterior and anterior pituitaries, respectively. *M. i*., massa intermedia; *T. M*., tractus Meynert; *V. d'A*. = mammillothalamic tract. **(E–G)**. These maps are from successive studies (Grossman, 1962; Booth, 1967; Leibowitz, 1978) attempting to resolve the effective stimulation sites where norepinephrine produces feeding. The first two maps **(E,F)** are plotted to the reference atlas of de Groot (1959a,b) and are directly comparable. In **(E)**, filled circles are indicative of positive effects of adrenergic and cholinergic stimulation; white stars indicate cholinergic effects; black triangles represent negative placements. In **(F)**, effective sites are solid stars; ineffective sites are open stars. In contrast, the sagittal map **(G)** furnished by Leibowitz (1978) is from the atlas of König and Klippel (1963). Numbers on the map in **(G)** indicate grams of food intake consumed following an injection in to that site. **(H)** Figure 3 of Stanley et al. (1993b) is partly reproduced and shown. The figure shows a portion of the Paxinos and Watson (1986) reference atlas, with locations of injection sites (*circles*) diagrammatically superimposed on a portion of the atlas plate. Numbers within the circles indicate grams of food intake following injection of a 10 nl solution containing 78 pmol of neuropeptide Y into the locations beneath each number. Note that the numbers on the left hemisphere (*in white circles*) correspond to 1 h food intakes following injection, whereas numbers on the right hemisphere (*in black circles*) correspond to 4 h food intake scores. **(I)** A sagittal map from the atlas of Paxinos and Watson (1998) showing effective feeding stimulation sites following injections of the μ-opioid agonist, DAMGO, into the nucleus accumbens by Peciña and Berridge (2005). The scale listed denotes the color scheme to show increasing magnitudes of food intake consumed. **(J)** Cannula placements within the ventral tegmental area where leptin injections were examined for their ability to reduce food intake. From Hommel et al. (2006). **(K)** A map of cannula placements for mice transduced with Channelrhodopsin-2 constructs within neurons expressing hypocretin/orexin, included as supplementary material for the study by Adamantidis et al. (2007). Optical fibers were inserted to stimulate these neurons and wake mice up from sleep. Blue squares denote animals with Channelrhodopsin-2 expression; red circles denote controls that displayed only reporter molecule expression (mCherry). **(L)** *Camera lucida* drawings of locations of hypocretin/orexin neurons in relation to injection sites locations where NMDA and AMPA stimulated food intake. From Li et al. (2011). **(M)** Injection sites in the vicinity of the ventromedial prefrontal cortex, from Mena et al. (2011), mapped onto Paxinos and Watson atlas space, where DAMGO injections triggered food intake. The legend shows the symbols and the amount of food intake denoted by each. **(N)** Distribution of ChR2-expressing AgRP neurons in mice optically stimulated to eat voraciously. The numbers of transduced neurons are plotted in relation to inferred stereotaxic coordinates to align their location with mouse brain atlas plates. From the supplementary data accompanying the report by Aponte et al. (2011). **(O)** Distribution of POMC neurons expressing the DREADD, hM4D in a *Pomc-Cre* mouse, from the study by Atasoy et al. (2012). The distribution is plotted along Bregma coordinates using inferred stereotaxic space. All figures are reproduced with permission of their original publishers, as noted here: Panels **(C** and **E)** (American Physiological Society); **(D)** (John Wiley & Sons, Ltd.); **(F)** (American Association for the Advancement of Science); **(G,H,L,M)** (Elsevier/Dr. Brian A. Baldo); **(I)** (Society for Neuroscience); **(J)** (Cell Press, Inc.); **(K,N,O)** (Nature Publishing Group).

Demole (1927) not only published some of the earliest photographs of brain injection sites (see Section 2.2), but also the first detailed “map” of sites in the cat hypothalamus where injections produced sleep (Figure 2A). This work also included what is possibly the first documentation of effective and ineffective injection sites in relation to gross anatomical landmarks on the ventral surface of the brain. The two types of site were mapped to opposite sides of the midline for diagrammatic purposes; stylistically, this use of mirrored hemispheres finds currency in later injection site maps (e.g., Stanley et al., 1993a,b,c). Comparing Figure 1A with Figure 2A, it is clear that although Demole cut brains in the coronal (transverse) plane, the “map” is in the horizontal plane. Therefore, the clearly subjective transformation involved to map the sites from one plane to another likely introduced a degree of inaccuracy.

Clöetta and Fischer (1930) supplied a similar map, albeit with fewer injection sites, for their injections of inorganic salts into the infundibular region of rabbits, rats and cats; to examine effects on respiration (Figure 2B). Like Demole (1927), they found that calcium chloride injections produced somnolence. However, Myers (1974) noted that their large injections (12–50 μl) likely resulted in their entry into the ventricles. Comroe (1943) performed an extensive study of the hindbrain respiratory center of 115 cats, each anesthetized in a stereotaxic frame so that chemical injections (bicarbonate, acids, potassium chloride, acetylcholine, nicotine, strychnine) could be documented in stereotaxic space. Rather than photograph each injection site (a task not feasible for so many subjects), Comroe reported the physiological outcomes of these experiments in the form of a functional map, shown in Figure 2C. Larsson (1954), who provided the first evidence that food intake can be controlled through chemical manipulations, also published the first map of effective feeding stimulation sites in the brain. In this study, the skull of the subject was first visualized by X-rays to record where the embedded steel needles were located. Effective injection sites lay posterior to the optic chiasm and lateral to the fornix (Figure 2D).

#### 2.3.2. Anatomical specificity and central injections

During the late 1960's and early 1970's, controversy surrounded ICI methods from the gradual realization in the scientific community that centrally injected carbachol and angiotensin II triggered drinking behavior that, in fact, was not necessarily due to actions within deep brain tissue sites, but because of actions through the ventricular system of the brain upon circumventricular structures such as the subfornical organ (Baxter, 1967; Myers and Sharpe, 1968; Montgomery et al., 1969; Epstein et al., 1970; Routtenberg, 1972; Singer and Montgomery, 1973; Johnson and Epstein, 1975; Epstein, 1978). Although space precludes detailed review of the shifting tides of this discussion during that period of time, the lessons learned from this controversy may be summarized as follows (see also Wise and Hoffman, 1992): (1) anatomical specificity of an injection site requires careful controls, especially since injected solutions can diffuse up and around the cannula needle and spill into the ventricular circulation if the needle has traversed through the ventricular compartment; (2) the injection volume will determine its likelihood of entry into the ventricular system, especially if injections are near the ventricles; (3) careful “mapping” experiments are necessary to provide a convergence of evidence that the concluded “site of action” for a substance is indeed the most sensitive site. This lesson is exemplified by the discussion in the next section concerning the locus of noradrenergic actions to produce feeding. As we shall see, careful cannula mapping studies were conducted in successive fashion by a few investigators to gradually refine the map of effective sites where NE injections triggered food intake.

#### 2.3.3. Mapping the feeding effects of norepinephrine (NE)

Following Grossman's (1960) report (see Section 2.2), the locations of hypothalamic loci effective for noradrenergic feeding were refined. Grossman (1962) published a more detailed set of data concerning the effects of carbachol and NE on feeding and drinking, respectively; which included a map of effective and ineffective injection sites (Figure 2E). These sites were plotted onto Jacob de Groot's stereotaxic rat brain atlas, first published for the whole forebrain in August of 1959, and then specifically for the hypothalamus in December of the same year [de Groot (1959a,b)]. The atlas plates for the hypothalamus were sampled at slightly better than twice the resolution of Wendell Krieg's 1946 atlas (the only other published for the rat brain at the time), and provided greater detail. (Apparently, Krieg had a more detailed rat brain atlas he had prepared in 1935, but which he had never published; see Krieg, 1975, p. 80). Importantly, Grossman (1962) set a new standard for mapping the effects of centrally injected compounds into the brain on food intake. Documentation using the de Groot atlas allowed for reproducibility and re-examination of the same brain regions by others with greater precision. However, as for the first report (Grossman, 1960), NE crystals were tapped into the cannula, preventing precise determinations of effective concentrations *in vivo* (see also Routtenberg, 1972; Wise and Hoffman, 1992).

In 1990, Booth, a biochemist in Neal Miller's lab, mapped the effects of centrally injected NE, dissolved in solution, on food intake. Locations plotted on de Groot plates allowed direct comparisons to be made with Grossman's (1962) study (Figures 2E,F). Booth showed that a hypothalamic region anterior and medial (A6.2, A6.6; Figure 2F) to Grossman's reported site (A5.4; Figure 2E) was the most sensitive site for adrenergic agonists to stimulate feeding. It is unclear whether Booth's injection volume (0.675 μl) delivered more or less NE than Grossman's crystal deposits did, but is perhaps a main reason why their results differ with respect to the site of action for NE. Indeed, data from Wagner and de Groot (1963) supported Grossman's finding that the lateral hypothalamus was a sensitive site, but large injection volumes (5 μl) were used. Using 1.0 μl NE, Slangen and Miller (1969) published histology (but not atlas plots) that generally confirmed Booth's anterior and medial locus for the feeding effects of NE, but also concluded that the perifornical region, in the vicinity of their injections, was likely to be a sensitive site. In contrast, Davis and Keesey (1971), who mapped their NE injections (0.5–1.0 μl) using the rat brain stereotaxic atlas produced by König and Klippel (1963) did not find the perifornical locus to be sensitive. Leibowitz (1978) published a systematic injection mapping study in over 500 animals across 35 separate locations within and outside of the hypothalamus. Using 0.5 μl of NE, she confirmed Booth's observations that the most sensitive site was in the medial and not lateral hypothalamus, and further provided compelling evidence that the area most sensitive was within the paraventricular nucleus of the hypothalamus (Figure 2G).

#### 2.3.4. Mapping the effects of peptides on feeding behavior

In Section 2.2, selected studies were highlighted that reported the acute control of feeding by peptide injections. Feeding stimulation sites have been mapped for opioids (Woods and Leibowitz, 1985; Stanley et al., 1988; Bakshi and Kelley, 1993; Peciña and Berridge, 2000, 2005; Smith and Berridge, 2005; Mena et al., 2011, 2013), H/O (Dube et al., 1999; Sweet et al., 1999); and AgRP (Kim et al., 2000), among many others. Similarly, the feeding-suppressive effects of several peptides have been mapped or at least partially localized, including bombesin (Kyrkouli et al., 1987); α-melanocyte stimulating hormone (Kim et al., 2000); cholecystokinin (Blevins et al., 2000); glucagon-like peptide 1-(7-36) amide (Schick et al., 2003), and pituitary adenylate cyclase-activating polypeptide (Resch et al., 2011). Below, NPY feeding stimulation is examined in detail.

***2.3.4.1. NPY and feeding: spatial considerations***. Stanley et al. (1993b) undertook an extensive cannula mapping study to identify hypothalamic sites sensitive to the orexigenic effects of NPY. Injections (78 pmol) were made in the very small volume of 10 nl into 47 brain regions in rats fed to satiety prior to injection, and food intake was measured 1 and 4 h post-injection. An area centered on the perifornical hypothalamus at the level of the caudal paraventricular hypothalamus was identified as a site where NPY exerted its most powerful effects on food intake, producing about 12.5 g of intake within the first hour. Additional sites near this locus also displayed strong sensitivity to NPY's orexigenic effects. Figure 2H shows some of these NPY-sensitive sites at the level of the dorsomedial hypothalamus (DMH).

**Figure 3:**
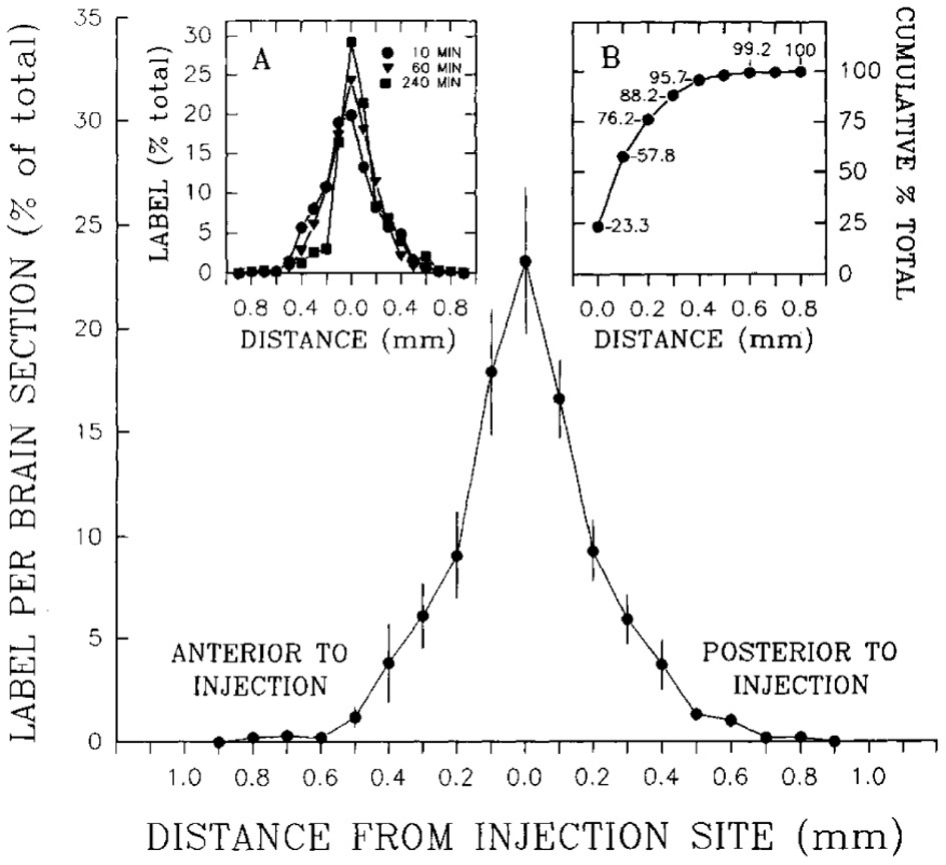
**A reproduction of Figure 2 of Stanley et al. (1993a,b,c), which describes the relationship between the percent recovery of radiolabeled neuropeptide Y (10 nl) within brain tissue 10 min following its injection into the brains of rats**. The percent recovery values are plotted against distance from the injection site in millimeters. Brain tissue sections were taken at 0.1 mm intervals from each injection site and the amount of radioactive NPY analyzed. The left inset in this figure (*labeled A*) shows the same relationship, but with 10, 60, and 240 min recovery times compared. The right inset (*labeled B*) shows the cumulative recovery of the radioactive peptide in relation to the distance at which the recovery was achieved; the numbers indicate cumulative percent recovery values. Note that the distance scale marks only the spread of the solution along the anterior-posterior axis, and does not speak necessarily to movement of the injected solution along any other dimensions. Reproduced with permission from Elsevier.

To determine how far 10 nl of injected solution spread to sites surrounding the injection site, Stanley et al. (1993b) also injected 10 nl of radiolabeled neuropeptide Y ([^125^I]-NPY) into the perifornical hypothalamus and tracked the anterior-posterior spread of the injected solution in sections taken at 100 μm intervals from the injection site. Radioactivity from these sections was measured as a function of both time and distance from the injection. In addition, an attempt was made to account for 100% of the radiolabel in the experiment by carefully measuring the radioactivity in the tissue as well as residual radioactivity associated with the guide cannula itself. Approximately 95% of the total amount of radiolabel was accounted for by its presence in the tissue and cannula, and 100% of the tissue label could be recovered at distances no more than 0.8 mm from the injection site. Importantly, 95% of this label spread to as little as 400 μm away from the injection site (Figure 3). As we shall see in Section 4.4.9, several approaches have been used to track the spread of an injected solution using various labeling strategies. However, this study has remained unique in using such small volumes to do so, and by using the same peptide that was producing the observed behavior. In Section 4, these unique data will be integrated within a spatial analysis of possible neural substrates underlying NPY-induced feeding behavior.

***2.3.4.2. Opioids and feeding: spatial and functional considerations***. Studies from the Berridge laboratory delineated novel feeding-sensitive subregions of the accumbens and ventral pallidum using a variety of carefully designed anatomical validation approaches. These were based on a novel functional approach to examine centrally injected chemical solutions first developed by Peciña and Berridge (2000), based on the detection of the transcription factor, Fos, in neurons following their activation by stimuli (Morgan et al., 1987; Sagar et al., 1988). The authors reasoned that although the diffusion of injected chemical solutions are difficult to track *in vivo*, and difficult to infer *post-mortem* from an injection site alone, they could track the functional activation of receptors at the injection site using Fos to mark neurons actively affected by the injections. Although the Fos transcription factor is a neuronal activation marker, its induction is not invariably correlated with increases in neuronal firing rates (Luckman et al., 1994; Watts et al., 2006). However, although this has not been examined systematically across several different brain regions, the association between Fos induction and neuronal activation is often assumed to be strong (Fos as a functional marker is discussed further in Section 4.4.8).

The authors used Fos immunohistochemistry to track neuronal activation in the injection site vicinity, and reasoned that by “subtracting” from their maps those Fos patterns that did not correlate with the appropriate behavior, they would isolate activation positively linked to the injections producing the behavior. From their behavioral experiments, they found that opioid receptor agonist injections into the shell of the nucleus accumbens triggered food intake. Morphine injections into this “eating site” also increased hedonic responses to an oral glucose solution, but failed to significantly change aversive responses to oral quinine infusions. “Fos plume” patterns marked a circumscribed area of the nucleus accumbens shell where hedonic reactions to sucrose were enhanced by intra-accumbens morphine. In a follow-up study, Peciña and Berridge (2005) furnished maps of the sites where hedonic and aversive reactions were affected by intra-accumbens injections of DAMGO, the synthetic enkephalin analog that is a μ-opioid receptor-selective agonist (Figures 1O, 2I). Importantly, for these studies, the authors were careful to point out that the Fos-expressing cells were not necessarily being activated directly by the injected opioids acting at opioid receptors on those cells. Indeed, evidence exists for both excitatory and inhibitory actions of opioids on neuronal excitability and synaptic transmission in the nucleus accumbens (Yuan et al., 1992; Chieng and Bekkers, 2001). Opioids are also capable of exerting disinhibitory actions by inhibiting interneurons synaptically interacting with local circuit neurons, which are thereby indirectly excited (e.g., see Zieglgänsberger et al., 1979). These caveats notwithstanding, the activation patterns did indicate that only a circumscribed set of neurons in the vicinity of the injection site was being recruited by the chemical injections.

Smith and Berridge (2005) used the Fos plume functional mapping procedure to examine the effects of opioids on feeding and reward mechanisms in the ventral pallidum. Injections of DAMGO had differential effects on hedonic “liking” reactions to sucrose, depending on whether the injections were centered in the posterior ventral pallidum (increased “liking”) or the anterior or central ventral pallidum (where “liking” reactions were suppressed). Similarly, DAMGO increased food intake in a parallel fashion, increasing feeding in the posterior ventral pallidum, but suppressing it when injected into the other subregions. Additionally, the authors found that injections of the GABA_A_ receptor antagonist, bicuculline, increased feeding at all ventral pallidum sites tested, but failed to enhance sucrose hedonic responses at any of them.

In addition to these findings, the authors extended the methodological approach of Peciña and Berridge (2000) in a number of important ways. First, the authors sought to define the boundaries of the ventral pallidum by immunohistochemically labeling the region using antibodies against leu-enkephalin in three planes of section, which is a peptide enriched in this region. They opted to document the immunolabeling patterns on a horizontal atlas level, since this plane of section includes a more or less complete representation of the ventral pallidum within one atlas section, thereby facilitating a simpler mapping template on which to plot all of their injections. Second, using the coronal atlas level of Paxinos and Watson that best fit with the section containing their injection site, the authors then defined the mediolateral and dorsoventral coordinates of the injection site off of the coronal atlas level axes (in mm). For the dorsoventral coordinate, the authors marked their injection sites with ink and then delineated the “center” of the injection site along the dorsoventral axis as the midpoint between the bottom of the microinjector needle and the furthest extent of the ink spread directly below the needle. In this fashion, they estimated sagittal and horizontal mappings for their coronal placements, in order to derive three-dimensional maps of injection site center points. For determinations of the anteroposterior atlas level, they noted cases that fell between atlas levels and chose the midpoint between two levels as the coordinate for the “between level” sections.

All of these approaches are noteworthy in that they impart to the reader the serious commitment the authors made in trying to document their placements to the best degree of certainty (for further details regarding the challenges of mapping these injection sites, please see Section 4 of Richard et al., 2013). A few additional improvements notwithstanding, these approaches stand out for the significance the authors place on experimental documentation as seen from the multiple sets of information provided: immunolabeling, ink deposits, Fos plumes, mapping along one plane of section rigorously, and extrapolations to additional planes of section. Minor “wish list” items that could have been added to these excellent approaches include: explicit delineations by symbols of those placements for which anteroposterior estimations fell between atlas levels (also see the concluding discussion of Section 4.6.1); tabulated data concerning the deviations of three dimensional physical coordinates for injection site centers from true, measured stereotaxic coordinates used to implant the cannulas; and a representative photograph of a brain section after ink has been injected. Overall, however, these studies constitute a signal example of how anatomical considerations can enrich any functional or behavioral study; indeed, the authors have set a higher standard by the thoughtfulness of their approach.

***2.3.4.3. Using phospho-STAT3 to track microinjected leptin***. Scientists have taken advantage of the fact that the adipose-derived hormone, leptin, activates specific signal transduction pathways with molecules that can be used as inducible markers of activation. For example, the spread of leptin following its microinjection into specific brain regions can be evaluated by monitoring the phosphorylation of STAT3 using a phospho-specific antibody raised against this molecule. Phospho-STAT3 labeling has been observed, for example, following injections of leptin into the VTA of mice (Hommel et al., 2006).

## 3. IGI methods: applications to feeding control and discussion of technical issues

### 3.1. IGI methods and feeding

Since the introduction of intracranial gene-directed strategies to drive expression of specific constructs into the brain using viral vectors, a number of investigators have used this approach to examine feeding and other motivated behaviors. Initial viral delivery strategies incorporated reporter molecules, such as β-galactosidase, to mark injection sites where virus was delivered (Davidson et al., 1993; Figure 1K). Table 2 highlights IGI methods used to study feeding control, including *in vivo* site-specific recombinase technology (Balthasar et al., 2005; Donato et al., 2011; Wu et al., 2012; Zhan et al., 2013) and viral-mediated expression or RNAi techniques. For example, Hommel et al. (2006) injected a construct that knocked down the leptin receptor gene, *Lepr*, in the VTA and produced altered feeding effects. The injection sites were carefully documented with respect to tyrosine hydroxylase-immunolabeled cell populations of the VTA and nearby substantia nigra (Figures 1Q, 2J), and also by phospho-STAT3 activation patterns (Section 2.3.4.2).

**Table 2:** **Transgenic technologies employed in selected IGI studies on feeding control**.

**Study**	**Species; construct; region**	**Effects observed**
**TECHNOLOGY 1: *in vivo* SITE-SPECIFIC RECOMBINASE TECHNOLOGY USING VIRAL INJECTIONS (e.g., cre-lox TECHNOLOGY)**		
Balthasar et al., 2005	Ms; MC4R; PVH	Re-introduced *Mc4r* in the PVH of transgenic mice lacking *Mc4r*, resulting in a ↓ BW gain
Hnasko et al., 2006	Ms; CAVCre; DA neurons	Restored feeding in DA-deficient mice by injecting CAV-Cre (Cre recombinase delivered by a canine adenovirus vector) into DA terminals in the central caudoputamen, which retrogradely transported CAVCre to TH^+^ cells in midbrain and restored TH expression in these neurons (reviewed by Palmiter, 2008).
Donato et al., 2011	Ms; *Lepr*; PMV	*Lepr* restored in PMV of *Lepr* KO showed no changes in food intake or BW, but ↑ puberty and ↑ fertility; also ↑ uterine weights, cross-sectional area, parenchyma size, and epithelial length; *Lepr* restored also normalized the GnRH content in the median eminence and mediobasal hypothalamus of LepR-null mice.
Wu et al., 2012	Ms; CAV2-Cre; *Tph2*; NTS	Blockade of 5HT_3_R signaling in NTS or *Tph2* inactivation in raphe neurons to NTS protects mice from starvation following AgRP neuronal ablation. Feeding and BW was also partially restored.
Zhan et al., 2013	Ms; hM3Dq, DtR ARH/NTS	Used Cre-inducible AAV vectors to deliver hM3Dq or DTR to POMC neurons of the ARH or NTS. See entry for Zhan et al. (2013) under “Pharmacosynthetics” (below), for details.
**TECHNOLOGY 2: VIRAL-MEDIATED EXPRESSION OR RNA INTERFERENCE**		
Dhillon et al., 2001	Rt; leptin; N/A (icv)	Delivered rAAV vector with cDNA for encoding Ob gene into the cerebroventricular circulation of rats. Found ↓ BW gain after two weeks without concomitant changes in food intake; found ↑ uncoupling protein expression in brown adipose tissue and a ↓ in white adipose content.
Szczypka et al., 2001	Ms; *TH/GTPCH*; CP	Delivered viral constructs (TH/GTPCH) for TH restoration in DA-deficient mice coupled with delivery of GTPC-I, which is essential for producing tetrahydrobiopterin, a cofactor critical for TH function. This mixed virus treatment in dorsal striatum restored feeding in KO mice (reviewed by Palmiter, 2008).
Li et al., 2003	Rt; *Pomc*; (ARH)	Overexpression ↑ hypothalamic *Pomc* in tissue homogenates, with a ↓ in food intake, and a moderate ↓ in BW gain; this was accompanied by a ↓ in visceral adiposity, fasting serum leptin, insulin, and cholesterol; and ↑ in uncoupling protein expression.
Kas et al., 2004	Rt; *Agouti*; PVH/DMH/LHA	PVH expression ↑ BW and food intake within 7 d; persisted >6 wk. LHA expression did not alter food intake or body weight in rats fed normal chow, but did in rats fed high-fat diet. DMH expression ↑ food intake and BW with a delayed onset (3 wk post-injection). No changes in AgRP, NPY, MCH, or Hcrt mRNAs.
Noordmans et al., 2004	Rt; GAD65; LHA	LHA overexpression ↓ BW gain and ↓ food intake, with no changes in water intake or feces production.
Hu et al., 2005	Ms; MCD; (ARH)	MCD overexpression ↑ food intake and BW; reversed feeding suppression caused by the fatty acid synthase inhibitor C75.
Hommel et al., 2006	Rt; Ob-R; VTA	Knockdown ↑ food intake, locomotor activity, and sensitivity to palatable food.
Keen-Rhinehart et al., 2005	Rt; Ob-R; hypothal (various)/DVC	ARH overexpression ↓ food intake; in all sites except the PVH, overexpression ↑ UCP-1 expression; estrous cycle was normalized and hypothalamic LHRH was ↑ following overexpression in ARH, MPOA.
Yang et al., 2006	Ms; SIM1; PVH	Knockdown ↑ food intake; overexpression ↓ food intake.
Dai et al., 2007	Ms; CPT1c; (ARH/VMH)	Overexpression ↓ BW gain following a high fat diet.
Gao et al., 2007	Rt; AMPK; (ARH)	Overexpression ↑ feeding after 3 d; ↓ i.c.v. leptin feeding and BW suppression; ↓ leptin inhibition of AMPK; blocked leptin activation of acetyl-CoA carboxylase (ACC), but not STAT3 induction; ↓ leptin from inhibiting AMPK and activating ACC and STAT3 in the PVH.
Garza et al., 2008	Rt; MC4R; PVH	PVH MC4R knockdown ↑ food intake and BW from a high-fat diet, but not after a normal diet.
McCrimmon et al., 2008	Rt; AMPKa; VMH	Knockdown ↓ glucagon, epinephrine responses to hypoglycemia by ~60 and 40%, respectively. More exogenous glucose needed to maintain hypoglycemia; endogenous glucose production and whole-body glucose uptake ↓. ↓ AMPKα associated with faster fall in glucose following insulin administration.
Yang et al., 2009	Rt; NPY; DMH	Overexpression in DMH of lean rats ↑ food intake, body weight and obesity brought on by a high fat diet; knockdown in DMH ↓ NPY innervation of the dorsal vagal complex and affected CCK satiation. *Npy* knockdown in OLETF rats ↓ basally elevated DMH *Npy* and ↓ BW, hyperphagia, glucose intolerance, insulin insensitivity, plasma leptin, and triglycerides.
Hayes et al., 2010	Rt; Ob-R; mNTS/AP	*Lepr* knockdown ↑ hyperphagia for chow, high fat and sucrose diets; ↑ BW/adiposity; ↓ sensitivity to CCK satiation; ↑ basal AMPK activity in mNTS/AP.
Tung et al., 2010	Rt; FTO; ARH/PVH	*Fto* overexpression in ARH ↓ food intake; knockdown to 40% of normal levels ↑ food intake; no BW changes observed; ↑ ARH gene expression for Stat3 and TH. *Fto* overexpression in the PVH ↓ food intake, but knockdown did not increase it.
Gao et al., 2011a	Rt; mutant CPT1; ARH	Mutant CPT1 overexpression prevented malonyl-CoA from inhibiting CPT1 but did not affect leptin-induced anorexia (see Dai et al. above).
Gao et al., 2011b	Rt; CPT1c; ARH	CPT1c overexpression ↑ feeding, ↑ ARH Npy and Bsx expression; ↓ leptin-induced anorexia and down-regulation of *Npy* and *Bsx* (see Dai et al. above).
Mineur et al., 2011	Ms; β4 nAchR; (ARH) + MC4R; PVH	β4 nACHR knockdown ↓ the anorectic actions of cytosine; PVH Mc4r knockdown ↓ nicotine-induced hypophagia, and cytosine-induced hypophagia.
Sousa-Ferreira et al., 2011	Rt; NPY; ARH	Overexpression ↑ diurnal overeating, BW, obesity; associated with elevated circulating leptin and dysfunctional adipocytes with no changes in POMC immunoreactivity; knockdown did not affect basal feeding or weight gain but ↓ fasting-induced hyperphagia and locomotion.
Nguyen et al., 2012	Ms; Y1 + Y5 NPY-Rs; PVH	Male and female mice with PVH-targeted knockdown of Y1/Y5-Rs had ↓ food intake to a high fat diet and various changes also seen in BW, depending on sex and condition.
Zhu et al., 2012	Rt; CCK1-R; DMH	Overexpression in OLETF rats had no change in feeding and BW; slight ↑ in daily food intake and significant ↓ in dark cycle meal size; and ↓ hyperglycemia.
Le Foll et al., 2013	Rt; FAT/CD36; VMH	Knockdown did not impair glucosensing, but ↓ number of VMH neurons exhibiting oleic acid responsiveness; no effect in weanlings on food intake, BW, or adiposity; but displayed ↑ plasma leptin levels, ↑ subcutaneous fat deposition, and abnormal glucose tolerance.
Zheng et al., 2013	Rt; NPY, DMH	Overexpression ↑ food intake caused by ↑ meal size during the dark phase, ↑ BW, and ↓ metabolic efficiency; ↑ high-fat diet-induced hyperphagia, obesity, and hyperinsulinemia.
**OPTOGENETICS**		
Aponte et al., 2011	Ms; ChR2; ARH	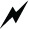 AgRP neurons ↑ feeding; 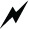 POMC neurons ↓ feeding, BW; feeding ↑ correlated with AgRP ChR2 transduction, but not with AgRP neuronal distribution; effect independent of melanocortin inhibition
Domingos et al., 2011	Ms; ChR2; VTA	Mice chose between licking from either a water feeder coupled to concomitant optogenetic DA neuron 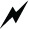 or one that dispensed artificial/natural sweeteners (sucralose and sucrose). Mice chose DA neuronal 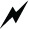 over sucralose but not sucrose. DA neuronal 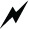 + sucralose, however, trumped choosing sucrose alone. Food restriction enhanced sucrose reward value over sucralose, whereas leptin reduced it.
Atasoy et al., 2012	Ms; ChR2, ARH/PVH	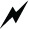 AgRP neurons ↑ GABA-mediated inhibition of POMC neurons; 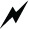 POMC neurons overcame AgRP sy^−^ evoked suppression of POMC neurons; co- 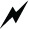 AgRP + POMC neurons ↑ feeding and latency to eat. 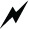 AgRP axons to PVH but not PBN ↑ feeding. AgRP axons to PVH ↑ GABA-mediated inhibition of PVH neurons. 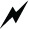 AgRP ↑ lever press break point. Co- 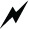 PVH AgRP
		axons + SIM1 neurons overcame AgRP inhibition of SIM1 neuronal activity; ↓ AgRP neuron-evoked feeding. AgRP axons ↓ PVH OT neurons. 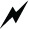 OT neurons ↑ Fos expression. Co- 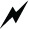 AgRP axons + OT neurons ↓ evoked feeding; 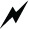 PVH OT neurons ↓ AgRP- 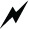 induced feeding. Blocking PVH GABA_A_-Rs, Y1-Rs ↓ PVH AgRP axonal 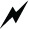 -evoked feeding.
Kong et al., 2012	Ms; ChR2, ARH/NTS	ChR2 expression mainly in PVH axons originating from RIP-Cre neurons in ARH; 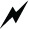 Rip-Cre axons in PVH triggered inhibitory post-synaptic currents, ↓ by bicuculline (GABA_A_ receptor antagonist). ChR2 axons comingled with ventral zone, medial parvicellular PVH neurons displaying fluorescent beads retrogradely transported from the NTS/dorsal motor nucleus of the vagus. IPSCs observed after 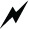 in retrogradely traced neurons. In contrast, AgRP neurons in the ARH transduced with ChR2 had axons that, when 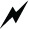 in the PVH, did not produce IPSCs in the traced neurons. 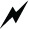 NTS neurons ↑ IPSCs in the raphe pallidus.
Calu et al., 2013	Rt; eNpHR3.0, dmPFC	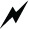 (−) dmPFC neurons ↓ stress-induced reinstatement of food seeking, Fos expression, and electrical activity; but not inflammatory responses in the mPFC.
**PHARMACOSYNTHETICS**		
Krashes et al., 2011	Ms; hM3Dq, hM4Di; ARH	CNO depolarized and ↑ firing rate of hM3Dq-expressing AgRP neurons in brain slices and ↑ Fos expression in the ARH. CNO ↑ food intake four-fold in transduced mice in the first 30 min post-injection, with a concomitant ↓ in energy expenditure lasting 8 h. Chronic stimulation using CNO ↑ BW, food intake. This effect wore off 5 d after withdrawal of CNO administration. CNO hyperpolarized and ↓ firing rates of AgRP neurons transduced with hM4Di, and ↓ food intake. AgRP-neuron stimulated mice also achieved higher break points in a nose-poke operant conditioning paradigm. 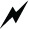 AgRP neurons in the absence of food ↑ “intense, unrelenting activity” lasting for hours, indicative of food-seeking behavior.
Atasoy et al., 2012	Ms; hM4D; ARH/PVH + PSAM-GlyRs; PVH + hM3D; ARH	The hM4D agonist, CNO, ↓ POMC neuron activity in slices, did not alter 1 h food intake *in vivo*, but ↓ 24 h intake. CNO ↓ SIM1 neuron electrical activity and ↑ food intake. PSEM silenced SIM1 neurons and ↑ feeding. SIM1 neuronal silencing ↑ lever press break point. CNO-mediated AgRP activation the ARH ↑ feeding that was ↓ by PVH-applied GABA_A_-R or NPY Y1-R antagonists but still above baseline.
Kong et al., 2012	Ms; hM3Dq, ARH	CNO depolarized hM3Dq-expressing ARH Rip-Cre neurons in brain slices; CNO ↑ ARH Fos in Rip-Cre mice, and Rip-Cre, Vgat^flox/flox^ mice. Selective activation of Rip-Cre neurons ↑ O_2_ consumption and brown adipose tissue activity, but not in mice unable to release GABA (Rip-Cre, Vgat^flox/flox^ mice) or those not expressing hM3Dq (Vgat^flox/flox^ mice); in contrast, there were no feeding effects following this treatment.
Zhan et al., 2013	Ms; hM3Dq, DTR; ARH/NTS	CNO depolarized POMC neurons expressing hM3Dq in the presence of TTX, as well as NTS neurons; Fos activated in roughly 50% of hM3Dq-expressing neurons in ARH and NTS after CNO application *in vivo*. Consecutive CNO injected over 3 d (but not acutely over 1 d) ↓ daily food intake and BW in mice with ARH hM3Dq expression. In contrast, acute activation of hM3Dq-expressing NTS neurons ↓ feeding (within 2 h), and ↓ meal number and meal size, and a second CNO injection prolonged this inhibition for 3 h more (albeit w/subsequent compensatory feeding). Locomotor activity, in contrast, remained unchanged. DTR expression in ARH ↑ obesity, BW and ↓ energy expenditure, ↑ body fat, ↓ lean mass, ↑ lipid profiles, hyperglycemia, and ↓ glucose tolerance. DTR expression in the NTS had no such effects.

*This is a list of selected gene-directed technologies that have used to examine the central control of food intake: in vivo site-specific recombinase technology; viral-mediated overexpression or RNA interference (RNAi); optogenetics; and pharmacosynthetics. Symbols used in this table: Middle column: species include mouse (Ms) and rat (Rt); brain regions include the AP, area postrema; ARH, arcuate nucleus of the hypothalamus; DMH, dorsomedial nucleus of the hypothalamus; dmPFC, dorsomedial prefrontal cortex; MPOA, medial preoptic area; NTS, nucleus of the solitary tract; with mNTS denoting the medial subnucleus of the NTS; PMV, ventral premammillary area; PVH, paraventricular nucleus of the hypothalamus; VMH, ventromedial nucleus of the hypothalamus or ventromedial hypothalamus; and VTA, ventral tegmental area. DTR, diptheria toxin receptor; hM3D, hM3dq, hM4D, and hM4Di are all DREADDs (see text for details). Right column: AMPK, AMP kinase; AgRP, Agouti-Related Peptide; BW, body weight; ChR2, Channelrhodopsin-2; CNO, clozapine-N-oxide; CPT1, carnitine palmitoyltransferase; d, days; DA, dopamine; FTO, fat mass and obesity associated gene; GTPCH, GTP cyclohydrolase; h, hours; Hcrt, hypocretin/orexin; IPSCs, inhibitory postsynaptic currents; MC4R, melanocortin 4 receptor; MCH, melanin concentrating hormone; nAchR, nicotinic acetylcholine receptor; OLETF, Otsuka Long Evans Tokushima Fatty rats; POMC, pro-opiomelanocortin; TH, tyrosine hydroxylase; TTX, tetrodotoxin; wk, weeks; ↑, produced an increase in a measured variable (e.g., food intake, uterine weights), stimulated a physiological response, or triggered a behavioral outcome; ↓, produced a decrease in a measured variable, inhibited a physiological response, or suppressed a behavioral outcome;* 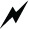
*, optogenetic stimulation; co-*
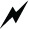
*, concomitant optical stimulation;* 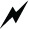
*(−), optogenetic silencing*.

More recently developed IGI methods include optogenetics and pharmacosynthetics (Table 2). The first demonstration of acute, optogenetically-driven behavioral control in a rodent model was reported by the de Lecea laboratory at Stanford University, who demonstrated that optogenetic stimulation of H/O neurons in mice was sufficient to wake them up from sleep (Adamantidis et al., 2007; see also Carter et al., 2013). Figure 2K shows their cannula placement map for mice transduced with Channelrhodopsin-2 (ChR2) in H/O neurons, and control rats transduced only with the reporter molecule, mCherry; plotted in relation to the subthalamic region and hypothalamus. Others have since used optogenetic methods to probe hypothalamic substrates to control other behaviors. For example, the Anderson laboratory delineated a ventromedial hypothalamic “locus” controlling aggression and documented their optical fiber placements [(Lin et al., 2011); reviewed by Anderson (2012)]. Konadhode et al. (2013) also showed that optogenetic stimulation of melanin concentrating hormone neurons increases sleep. Nieh et al. (2013) have presented a cogent summary of recent optogenetic work demonstrating a role for key loci throughout the brain in controlling motivated behaviors.

In relation to feeding behavior, a few studies using optogenetics and pharmacosynthetics have now been published (Table 2), a few of which will be noted here. Aponte et al. (2011) inserted ChR2 constructs into AgRP-containing neurons within the mouse arcuate hypothalamus. Activation of the transduced neurons triggered a robust feeding response. In their study, an optical fiber placement was documented over transduced neurons (Figure 1T), and maps of the mouse brain were provided upon which the total numbers of neurons transduced with ChR2 were indicated. A summary of these cell counts was also plotted with respect to Bregma coordinates from the corresponding atlas plates (Figure 2N), suggesting the use of *inferred stereotaxic space*, a concept discussed in Section 4.6.2.1.1. A similar strategy was employed for an elegant follow-up study from the same laboratory [(Atasoy et al., 2012); reviewed by Sternson (2013); Sternson et al. (2013); see Table 2]. Specifically, the authors showed that optically stimulated ChR2-transduced AgRP neurons drive feeding responses through inhibition of oxytocin-containing neurons of the paraventricular hypothalamus. They showed further that the synthetic and brain-penetrable ligand, CNO, could produce long-term inhibition of POMC neurons transduced with hM4D, a DREADD (Designer Receptor Exclusively Activated upon by a Designer Drug); and that this inhibition also increased feeding. The authors provided distribution data for the rostrocaudal spread of their transduced POMC population (Figure 2N). Although ChR2-transduced POMC neurons are Fos-activated by optogenetic stimulation (Figure 1U), the authors did not report the rostrocaudal distribution of the Fos-activated neurons, which ostensibly would be a subset of the ChR2-expressing population. In another elegant study, the Lowell laboratory showed Fos activation in pharmacosynthetically activated AgRP neurons (but without a mapped distribution) that was accompanied by robust feeding (Krashes et al., 2011). Their approach showed, as did the optogenetic studies, that AgRP neuronal activation was sufficient to trigger feeding behavior. Finally, Calu et al. (2013) recently used optogenetic neuronal silencing to examine feeding control by targeted a light sensitive halorhodopsin bilaterally to the dorsal medial prefrontal cortex and examined stress-induced reinstatement of palatable food seeking in female rats.

### 3.2. Methodological issues concerning anatomical documentation for IGI methods

#### 3.2.1. Virus transduction efficacy and variability

One important consideration with IGI studies is the extent to which a neuronal population that is being targeted by a virally delivered construct has successfully been transduced with this construct. In rat brain, for example, where the tissue expanse is larger than that of a mouse brain, multiple injections of a lentivirus or adeno-associated virus typically are employed in order to effectively deliver virus to broadly distributed cell populations. In such cases, mapping the successfully transduced neurons is an important documentation procedure that aids readers in independently evaluating the effects of the gene-directed delivery, whether it is for optogenetic stimulation or silencing, or knockdown or overexpression of a particular molecule. In their seminal study establishing the feasibility of adenoviral vector mediated delivery of a transgene into the brain, Davidson et al. (1993) used β-galactosidase as a reporter construct within their vector; the staining for which showed the extent of viral transduction in the brain (Figure 1K). Similar approaches have been used for other IGI studies. For example, in their study (Abbott et al., 2011) of the effects of optogenetic stimulation on Phox2b-expressing neurons in the rat retrotrapezoid nucleus, which produced an activation of multiple components of breathing (respiratory rate, inspiration, expiration), Patrice Guyenet and colleagues not only carefully mapped the locations of all of their ChR2-expressing neurons across various hindbrain levels, but did so for all twelve subjects they were examining (see their Figure 8). This approach nicely illustrates the careful documentation of transduction efficacy that also takes into consideration inter-subject variability.

#### 3.2.2. Promoter specificity and strength

Yizhar et al. (2011) discuss the types of promoter fragments that have been used for optogenetic studies. The use of strong promoters over a long period of time may result in ChR2 expression being associated with abnormal axonal morphology and changes in the targeting of axons to particular locations (Miyashita et al., 2013).

#### 3.2.3. Methodological considerations unique to optogenetics or pharmacosynthetics

***3.2.3.1. Optogenetics: inferring connections originating from single neurons within a stimulated population, and c-Fos activation***. A consideration especially important for optogenetic applications concerns the degree to which one can gauge the selective recruitment of transduced neurons following *in vivo* light stimulation. Just because a group of neurons has been transduced successfully for ChR2 gene expression does not mean they all fire with equal fidelity to optical pulses. Those that do may be intermingled and co-distributed with those that do not. Are the axonal connections for all optically stimulated or unstimulated neurons the same with respect to target neurons? The outputs of subsets of the activated, driven cells may be differential with respect to the outputs of other subsets. In this regard, Fos activation studies *in vivo* following light pulses could help to delineate activated cells and allow one to infer subpopulations that may be involved. The data from Aponte et al. (2011) and Atasoy et al. (2012), for example, demonstrate that a population of arcuate hypothalamic (ARH) neurons expressing the neuropeptide AgRP are sufficient to orchestrate robust feeding behavior. The rostrocaudal distribution of the Channelrhodopsin-expressing neurons is known from their studies, but the specific neurons along this axis that were Fos-activated and that produce specific feeding responses is unclear. Knowing the locations of these neurons through the rostrocaudal extent of the arcuate hypothalamus would be very beneficial so as to understand their spatial relation with other cell populations in the hypothalamus. Similarly, the delineated distribution and mapping to an atlas of the CNO-activated subset of Fos-labeled arcuate neurons in the report by Krashes et al. (2011) would allow others to interrelate those data with the anatomical locations of activated neurons in their own experiments.

***3.2.3.2. Optogenetics: activating labeled axons***. Atasoy et al. (2012) identified a pathway from mouse ARH neurons to oxytocin-expressing paraventricular hypothalamic (PVH) neurons with descending connections to the hindbrain. Similarly, Kong et al. (2012) identified GABAergic “RIP-Cre” neurons in the mouse ARH that control PVH neurons with descending projections to the nucleus of the solitary tract to selectively control energy expenditure without concomitant changes in food intake [reviewed by Bouret (2012)]. These studies demonstrated behavioral or autonomic changes brought about by selective stimulation of descending PVH connections. Mendelowitz and colleagues have highlighted how strong promoters are required to drive ChR2 expression in descending axonal projections originating from pre-autonomic PVH neurons (Piñol et al., 2012). It remains possible that weak or inefficient expression of a reporter molecule accompanying ChR2 expression in axons (e.g., EGFP) may also result in an incomplete labeling of all axons arising from transduced neurons. Thus, although reporter expression in tissue sections distal to the location of transduced cell bodies may allow for selective fiber-delimited stimulation or silencing, the labeling of such projections may not reflect the complete set of fibers that arise from a transduced population. Ideally, one should have data regarding the locations of axonal projections of a particular group of neurons *prior* to designing an optogenetics experiment.

***3.2.3.3. Optogenetics: spatial disposition and penetrance of light***. Another issue related to optogenetic experiments is the need for the experimenter to estimate the degree to which light is penetrating deep tissue locations such as those found in the hypothalamus. Recent modeling of ChR2 mediated action potential generation in cortical pyramidal cells, for example, demonstrates that the threshold for action potential generation is highly dependent, in part, on light irradiance (Foutz et al., 2012). Using a 20-mW 488-nm diode laser on mouse tissue slices containing lateral hypothalamic neurons transfected with ChR2, it was estimated that 0.5 mm^3^ of tissue receives light of at least 1 mW/mm^2^ (Adamantidis et al., 2007). Laser light delivered by conventional optical fibers escapes as a cone of illumination with an intensity that attenuates as a function of distance the light travels from the fiber tip. Recently, μ-iLEDs have been shown to provide a more uniform, omnidirectional light delivery than conventional optical fibers; μ-iLEDs can also be fabricated for directionally selective light delivery [see Figure 3B in Kim et al. (2013b)]. Light penetrance in optogenetic applications involving deep brain stimulation has been addressed at the level of brain tissue itself. Specifically, the light scattering properties of mouse brain regions have been estimated *in vivo*, and a mouse brain atlas and accompanying software application (http://www.optogeneticsapp.com) have been created to assist researchers in designing their optogenetic experiments in a manner that ensures adequate light penetrance for their target brain region (Al-Juboori et al., 2013).

***3.2.3.4. Optogenetics: thermal changes associated with light pulse delivery***. Blue light laser stimulation to drive ChR2-mediated depolarization in transduced neurons may give rise to thermal confounds. Recently, for example, Christie et al. (2013) reported that rat cortical surface temperatures increased during blue light (445 nm) laser stimulation, with linear rises in temperature (0.5–7°C) for light transmitted at powers ranging from 0.6–16 mW, respectively.

#### 3.2.4. Summary of technical issues related to using IGI methods for feeding control studies

To recapitulate the main points of Section 3.2, the locations of probe placements, transduced neurons, and activated transduced neurons are all important to document as thoroughly as possible in IGI studies. Documentation about light and heat penetrance for optogenetics studies is also warranted. Documentation is also important for electrophysiologically recorded neurons labeled after validation assays using tissue slices *ex vivo* (often used in conjunction with IGI methods), which can be mapped if they are dye-filled or labeled in other ways in relation to the total population of surrounding neurons. Finally, atlas-based mapping of circuits probed using IGI methods is fundamental to establishing legacy datasets upon which to build further discoveries. As resources such as Cre recombinase-driver rats (Witten et al., 2011; see also Sato et al., 2004), certain ChR2-transgenic rats (Ting and Feng, 2013), and tools such as Brainbow adeno-associated viral vectors (Cai et al., 2013) gain more widespread use, then the lessons we have learned so far using rat models will also remain applicable to these newer technologies.

### 3.3. A note about ICI and IGI methods, feeding control, and “chemical coded behavior”

Before we turn to the next section, which is focused on applying concepts derived from functional neuroanatomy to feeding control, it is useful to interrelate ICI and IGI methods in terms of how they have helped to shape our understanding concerning general aspects of feeding behavior, such as the way behavior is coded by complex chemical circuits.

From the early applications of ICI methodology to feeding control described in Sections 2.2 and 2.3.3, emerged the concept of the “chemical coding of behavior,” which posits that distinct behaviors can be linked to actions of different chemicals acting within the same or different brain locations (Grossman, 1960; Miller, 1965). Despite the many benefits derived from using ICI methods, it is important to note the limitations of such approaches insofar as what they can tell us about the *endogenous operation* of chemical networks in the brain. For example, in the past 25 years, the field of ingestive behavior has generally paid closer attention to peptides acting to control food intake rather than small neurotransmitters; in part, because peptides are easier to localize than small transmitters in *post-mortem* tissue, and many have been shown (see Section 2) to acutely stimulate or suppress feeding following central injection. However, it appears from electrophysiological work that the primary ongoing synaptic activity for the endogenous operation of hypothalamic and other networks controlling feeding is driven by small neurotransmitters such as GABA and glutamate, and that peptides may exert a neuromodulatory function superimposed upon their continuous actions (see van den Pol, 2003, 2012; Marder, 2012). Thus, the results of behavioral and electrophysiological experiments, along with the interpretations of these experiments by many investigators, appear to differentially emphasize certain elements of chemical space and their importance with respect to the endogenous operations of circuits participating in feeding control. Nevertheless, scientists have drawn conclusions about the endogenous or native chemoarchitecture of the hypothalamus from the effects they observe when certain chemicals, including proteins, are injected. It remains to be seen whether this “chemical coding” is a concept that is incorrect, or correct but inaccurately and/or incompletely determined by ICI methodology [see, for example, the criticisms by Booth (1990), p. 474].

Moreover, while it is still too early to ascertain whether newer IGI methods will completely resolve this issue, they are already clarifying aspects of it in relation to feeding control. For example, recent IGI studies (e.g., Atasoy et al., 2012; see Section 3.1) have identified only a role for GABA co-localized within AgRP neurons in controlling food intake, but not necessarily AgRP itself. In contrast, central injection data indicate that AgRP can trigger feeding stimulation (Rossi et al., 1998; Hagan et al., 2000; Kim et al., 2000; Semjonous et al., 2009). Thus, the roles in feeding control for GABA, AgRP, and NPY (all normally produced within AgRP neurons) have been relatively unclear. This matter was addressed in an elegant study by Krashes et al. (2013), in which IGI methods were used to insert DREADDs (see Sections 1.1 and 3.1) in AgRP neurons of the arcuate hypothalamus within mice harboring single, site-specific deletions of genes encoding for NPY, GABA, or AgRP transmission, or in mice that had triple-gene deletions or bore double gene deletions (to remove GABA and NPY). The authors found that CNO-mediated stimulation of these animals resulted in food intake that appears to rely differentially on all three molecules: NPY or GABA are required for rapid feeding and can compensate for each other's absence, whereas AgRP can only mediate more prolonged, chronic feeding. The temporal differences in the actions of these molecules help to explain their roles in orchestrating feeding behavior over short and prolonged time scales. This is one example of how an IGI method (pharmacosynthetics), in combination with other transgenic methods, can help validate and clarify the data produced by ICI and other IGI (optogenetic) methods to advance our understanding of the role of various chemical constituents within neural circuits controlling feeding behavior. In the next section, we will examine how thinking about functional neuroanatomy in the context of ICI and IGI methods can further broaden and deepen our knowledge and understanding of feeding control.

## 4. A conceptual framework for thinking about feeding control sites in the brain

### 4.1. Central questions from central injections

In Sections 2 and 3, a review of some of the major central injection studies examining food intake was provided, with an emphasis placed on those studies that have included anatomical documentation in the form of injection site locations or maps of such locations. In this section, we discuss these studies in relation to a wider view of how injection sites and the neural substrates controlling feeding and other motivated behaviors can be contextualized in relation to neuroanatomy. Anatomical considerations are critical in answering three fundamental questions that arise from the targeted manipulation of neural substrates controlling food intake (or any other behavior for that matter). These questions are:

1. where is the injection site?
2. what (and where) are the neural substrates that are affected by the injected substance?
3. which, if any, of the affected neural substrates contribute to the behavior(s) observed?

In evaluating how these questions can be answered using knowledge of neuroanatomy, it is useful to remember that the answers to these questions (location of the injection site, the location and identity of the cellular machinery affected by the injections, and the subset of such machinery that contributes to the observed behaviors); all reside within the brain. The starting point to answer all of these questions, therefore, resides in analyzing the physical substance of the manipulated brain. Traditionally, this has usually been conducted *post-mortem*, although non-invasive functional magnetic resonance imaging (fMRI) of the living brain in the intact organism has been performed following pharmacological manipulations known to affect feeding (Stark et al., 2006, 2008; Dodd et al., 2009, 2010). Modified fMRI and positron emission tomography (PET) methods have also has been used to obtain real-time data confirming the efficacy of *in vivo* optogenetic stimulation (Domingos et al., 2011; Thanos et al., 2013; but see Christie et al., 2013). The state-of-the-art for these applications, however, is not yet at cellular resolution.

Remembering that the physical brain itself is the object of analysis is, on one level, rather obvious. But on another level, the concept acquires profundity, especially when one asks if all of the questions posed above for a particular study can be answered by *post-mortem* analysis of a *single* subject's brain. Does visualizing the injection site in a selected brain specimen, for example, *methodologically preclude* one from examining the affected neural substrates in the same tissue? If so, then one must resort to examining certain animals from a given cohort for particular data, some for the injection site, and others for a view of the affected neural substrates. But if this latter approach is used, how does one compare histology from different brains to interrelate these two distinct sets of data? The questions, then, are reduced down to those concerning histology; it is therefore worth examining what happens in this process more closely.

### 4.2. Localizing injection sites

“*The gains in brain are mainly in the stain*.”

*—Floyd E. Bloom (1975)*

Bloom's famous adage, which is a useful starting point to begin our discussion of histological matters, informs us that our understanding of the structure of the brain is dependent on the methodologies used to visualize it. The type of histology performed will, *to varying degrees*, provide information about the location of the intracranial manipulation and/or the neural substrates affected by such a manipulation. Consider, for example, how the studies outlined in Sections 2 and 3 were conducted. The investigators who performed these studies localized their injection sites and probe locations in various ways. One approach was through the use of gross anatomical landmarks, either provided by the appearance of the skull prior to injection, or from within the brain tissue itself. For example, recall that Demole (1927) used dyes such as ammoniacal carmine, Immedial black or India ink to mark injection sites within the living brain and examine these sites *post-mortem* (Figure 1B). In contrast, von Euler and Holmgren (1956) took X-rays of their cannula implants in the skull (Figure 1D) and others (Epstein, 1960b; Grossman, 1960) used a Nissl stain to see the injection near neighboring groups of neurons (Figures 1F,G).

### 4.3. “Visualizable spaces” of the brain: an introduction

Section 4.4.9 provides more information concerning the diverse ways in which injection sites have been localized (see Akert and Welker, 1962 for similar issues for electrode probes). From these few examples, however, emerges the general principle that the brain can be visualized in a variety of ways and each of these ways requires its own special methodology. Although we have just examined how a few scientists have tried answering the first of the three questions posed earlier (where is the injection site?), this principle requires further delineation to help address this question more fully and to also address the other two questions which do not concern the location of the injection site, but deal instead with the neural substrates affected by the injection.

**Figure 4:**
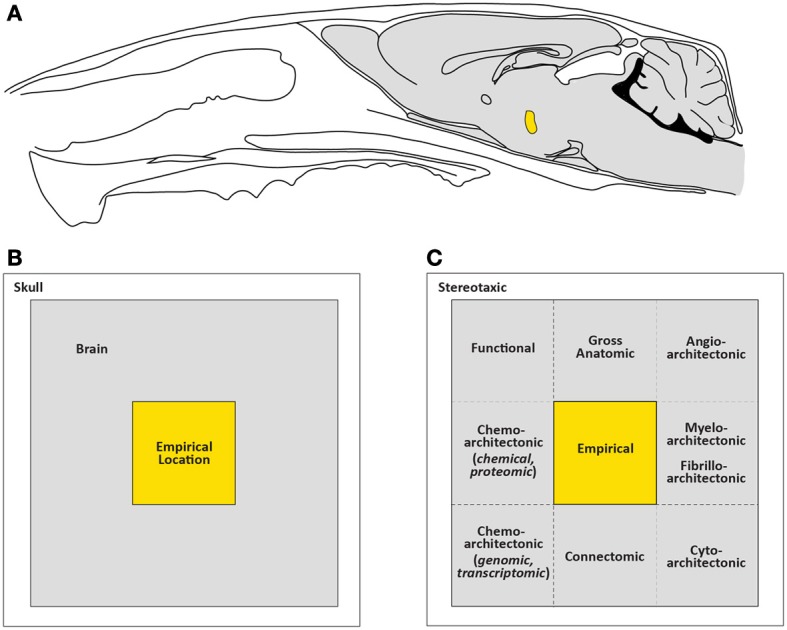
**A conceptual framework for organizing thoughts concerning injection site localization**. **(A)** Outline of a rat skull (black outline only), rat brain (gray), and injection site (gold); showing the topological relations of these three spaces. The drawing is adapted from König and Klippel (1963). **(B)** The topological relations shown in **(A)** are simplified in a nested square arrangement here. Note the names given to each space. **(C)** A conversion of the relationships presented in **(B)** within the context of methodologically visualizable spaces. Stereotaxic space is derived from skull measures and is therefore listed in the space corresponding to the location of the skull. This topological relationship affords stereotaxic space with the privileged position of being a reference for all other spaces. The space occupied by the brain can be visualized as any of the spaces listed. Dotted lines signify that each space is not the only one visualizable; but several can be detected simultaneously, depending on the compatibility of methods used. See text for further explanations.

Figure 4 illustrates a simple formalization of the ideas just discussed. In panels A and B, basic topological relations are shown for the skull, the brain it contains, and empirical location (injection site, for example) within the brain. Figure 4C then defines the brain as the sum of all possible “*visualizable spaces”* contained within it. Many of these visualizable spaces are *topologically distinct*, that is, it is not possible to interconvert them. Thus, an arterial lumen is topologically outside of the brain tissue proper, even though the vessel may be coursing inside the three-dimensional volume of the brain mass itself. This topological relation is analogous to a finger being carefully and slowly pushed against the taut skin of a gas-filled, transparent balloon: the finger is outside the balloon but might eventually be seen in “the center” of the balloon's dimensions. Thus, *angioarchitectonic space* is one visualizable space that is topologically outside the brain but physically still contained within its dimensions. Although the unique structural features of topologically distinct visualizable spaces preclude them from being interconverted, they all share the common feature of residing within the brain itself. Other spaces shown in Figure 4C are topologically within the brain and potentially interconvertible with other spaces. For example, *functional space* is such a space, since it can incorporate elements belonging to other spaces, depending on the functional marker used (protein, mRNA, etc.). Finally, many of these visualizable spaces are *conceptually indistinct*. Thus, blood vessels in the brain will inform both *gross anatomic* and *angioarchitectonic* spaces. Fos expression will inform both *functional* and *chemoarchitectonic (proteomic)* spaces, and so on.

### 4.4. “Visualizable spaces” of the brain: a closer look

Before we explore how visualizable spaces can help us organize our thinking about anatomical localization and registration in simple ways, it is important to define them more precisely. As will become evident from the introduction of each of these spaces below, technological developments over the years have allowed the visualization of certain neural elements to extend from one visualizable space to another. Table 3 highlights details for studies mentioned in this section and includes additional references related to each visualizable space as it has been studied in relation to feeding control.

**Table 3:** **The development of visualizable spaces and their use in ICI and IGI studies**.

**Gross anatomic space**	
•	Reviewed in Clark et al. (1938); see also Christ (1969), Nauta and Haymaker (1969).
**Angioarchitectonic space**	
•	Scremin (1970) mapped essential cerebral vessels to stereotaxic space.
•	Clark (1938), Finley (1940), Haymaker (1969), Ambach and Palkovits (1979): All are excellent reviews of hypothalamic angioarchitecture.
**Myeloarchitectonic and fibrilloarchitectonic spaces**	
•	Weigert (1882) developed stains specific for the myelin sheath (also see Swanson, 2000a).
•	Ganser (1882) first described the medial forebrain bundle (*basales Längsbündel)* in the mole.
•	Klüver and Barrera (1953) used Luxol Fast Blue, a sulfonated copper pthalocyanin salt, to stain myelin sheaths.
•	Wolf and Yen (1968) combined the Klüver–Barrera stain with Nissl methods (see also *cytoarchitectonic space*).
•	Nieuwenhuys et al. (1982) described the myeloarchitecture of rat medial forebrain bundle.
•	Miller et al. (1994), Saeki et al. (2001), and Lemaire et al. (2011) imaged myelinated fibers in human hypothalamus with magnetic resonance or diffusion tensor imaging (MRI/DTI). DTI is based on restricted H_2_O diffusion in such fibers (Douek et al., 1991).
**Cytoarchitectonic space**	
•	Golgi (1873) developed the “black reaction,” which allowed visualization of nerve cells in their entirety for the first time (see Pannese, 1999).
•	Nissl, in the 1890s visualized ribonucleic acids, allowing global visualization of cellular elements for the first time (see Nissl, 1913).
•	Brodmann (1909) established detailed parcellation of cortical structures using Nissl stains. Nissl-based atlases of hypothalamus include Christ (1969) and Bleier et al. (1979).
•	Golgi studies of hypothalamus include [see Millhouse (1979), for one overview]: arcuate nucleus (van den Pol and Cassidy, 1982); paraventricular nucleus: Armstrong et al. (1980); cells associated with the medial forebrain bundle at the level of the hypothalamus (Millhouse, 1969; McMullen and Almli, 1981)
•	van den Pol and Gallyas (1990) introduce a new esterification-silver procedure to induce Golgi-like staining in area of the hypothalamus subjected to light or severe trauma: light pressure on the ventral surface of the brain is sufficient to induce impregnations of neurons in several hypothalamic regions. The ventromedial hypothalamus has been observed using Golgi techniques to apparently undergo dendritic remodeling after food deprivation (Flanagan-Cato et al., 2008).
•	Viruses are developed as tools to produce Golgi-like staining in hypothalamic neurons (van den Pol et al., 2009).
•	Variants of the Golgi stain combined with fine histological sampling have been used to provide high-resolution views of the entire brain that allow for the brain to be “reconstructed” in other planes within three-dimensional space at cellular resolution (e.g., Li et al., 2010; Amunts et al., 2013).
•	Glial distribution in the hypothalamus has been reviewed by Polak and Azcoaga (1969). See related reviews by Hatton (2004) and Theodosis et al. (2008).
•	Non-neuronal cell types and their interactions within hypothalamus have also been documented (e.g., Mercier and Hatton, 2000, 2001; Mercier et al., 2003).
**Connectomic space**	
•	Waller (1850) unilaterally severed specific frog cranial nerves, causing their degeneration distal to the injury several days after lesion was made; linked nerve connection to origin of experimental injury.
•	Weigert (1882) applied his myelin stain on degenerating axons.
•	Marchi and Algeri (1885) developed a method to stain degenerating myelin selectively.
•	Frustrated that the Marchi–Algeri method precludes visualizing unmyelinated fibers (see Nauta, 1993), Nauta and Ryan (1952) developed a reduced silver stain for degenerating axons rather than degenerating myelin, extending *myeloarchitectonic space* to *fibrilloarchitectonic* and *connectomic spaces*.
•	Taylor and Weiss (1965) and Lasek (1967, 1968a,b) made seminal observations about axonal transport using radiolabeled amino acids.
•	Cowan et al. (1972) used radiolabeled amino acid injections and autoradiography to visualize anterogradely transported, radiolabeled proteins in non-degenerating axons and terminals.
•	Kristensson et al. (1971) used horseradish peroxidase to trace connections in either the anterograde or retrograde direction. The method could be combined with others to yield separate visualizable spaces in the same tissue (see Steward, 1981). Veening et al. (1982) used the method of Cowan et al. (1972) to identify the topography of the rat medial forebrain bundle, extending *myeloarchitectonic* data about this complex fiber system to *connectomic* space.
•	Gerfen and Sawchenko (1984) reported that the kidney bean lectin, *Phaseolus vulgaris*-leucoagglutinin (PHA-L) was transported primarily in the anterograde direction, could be deposited in very small volumes, was resistant to degradation, and labeled cells, axons and terminals equally well. Moreover, PHA-L could be used with other tissue processing methods to help visualize *connectomic* space with other spaces (Sawchenko and Gerfen, 1985; Wouterlood and Groenewegen, 1991). For details about tract-tracing, see the excellent volumes by Heimer and RoBards (1981); Heimer and Záborszky (1989), and Záborszky et al. (2006).
•	White et al. (1986) reconstructed serial electron micrographs from all 302 neurons defining the *C. elegans* nervous system (also see Brenner, 2003). This massive undertaking is an example, rare at the present time, of how microscopic anatomy can be used to inform connectomic space (for a discussion of the Brenner lab's achievement, also see Seung, 2012).
•	Petrovich et al. (2005) combined Fluorogold injections in the lateral hypothalamic area with *Arc* and *Homer1a* gene expression induced by a conditioned learning paradigm to identify the projections of neurons activated by cue-induced feeding (reviewed by Petrovich, 2011).
•	Coolen et al. (1999) developed a co-injection protocol for the simultaneous visualization of anterogradely and retrogradely labeled axonal connections in the brain; Thompson and Swanson (2010) further elaborated on this strategy by employing two sets of co-injections in the same experimental subject to delineate closed-loop network architecture in the brain.
•	Virus-based transneuronal tracers label polysynaptic pathways (Martin and Dolivo, 1983; for reviews, see Loewy, 1998; Card and Enquist, 1999); these have been used, for example, to trace sympathetic outflow systems from the paraventricular or lateral hypothalamus (Buijs et al., 2001; Oldfield et al., 2002; Geerling et al., 2003; Krout et al., 2003), and have been used in combination with electrophysiological recording to identify pre-autonomic neurons responding to peptides implicated in feeding and metabolic control (Davis et al., 2003). Conditional expression strategies used in combination with such tracers have been used to demonstrate afferents to the arcuate hypothalamus (DeFalco et al., 2001), efferent connections from the dorsomedial hypothalamus (Gautron et al., 2010), and pre-autonomic network connections involving the hypothalamus (Card et al., 2011; Lindberg et al., 2013).
•	Cai et al. (2013) have reported the development of Brainbow adeno-associated viral vectors for incorporation of Brainbow XFP cassettes into neurons by intracranial injection in species other than mice.
**Chemoarchitectonic space (chemical, proteomic)**	
•	Forssburg assisted Larsson in analyzing ^32^P incorporation into punched hypothalamic extracts from hungry and fed goats (see Part II of Larsson, 1954).
•	O'Donohue et al. (1979) mapped brain micropunch locations for α-melanocyte-stimulating hormone immunoreactivity.
•	Brownstein and Palkovits (1984) mapped brain micropunch locations for radioenzymatic analyses of neurotransmitters. Khan et al. (2004) examined tyrosine phosphorylation changes in hypothalamic tissue microdissected immediately following central injections of glutamate receptor agonists.
•	Morton et al. (2009) and Li et al. (2011) detected cell phenotypes near injected sites in the ventral tegmental and lateral hypothalamic areas.
**Chemoarchitectonic space (genomic, transcriptomic)**	
•	Segal et al. (2005), Yue et al. (2006), and Lee et al. (2012) used laser-capture microdissection/expression profiling to detect hypothalamic transcripts.
•	Khan et al. (2007) demonstrated central injection-induced activation of hypothalamic transcripts.
•	Watts (1991), Ponzio et al. (2007), and Khan et al. (2011) used intron-based PCR/hybridization to detect hypothalamic hnRNA after stimulation.
•	Food deprivation-induced gene expression changes in the hypothalamus have been analyzed using microarrays and/or quantitative PCR (e.g., Ding et al., 2010; Higgins et al., 2010; Poplawski et al., 2010).
•	Single-cell transcriptomics of electrophysiologically identified hypothalamic neurons has also been demonstrated (see Eberwine and Bartfai, 2011).
**Functional space**	
•	Gomori, in the 1930s, used histology to track regional brain activation patterns (see Gomori, 1952; van Noorden and Frederiks, 1992).
•	Hess (1956) and Thompson (1978) created atlases of those probe sites that produced gain or loss of behavioral function in cats and rats, respectively.
•	Sokoloff et al. (1977) used ^14^C-2-deoxy-d-glucose uptake and autoradiography to track regional activation patterns of the brain.
•	Morgan et al. (1987) and Sagar et al. (1988) developed detection methods for immediate-early gene (e.g., *c-fos*) activation.
•	Davidson et al. (1993) employed adenoviral vector gene delivery into the brain, utilizing a functional reporter gene (β-galactosidase) to mark the extent of the injection site and viral spread in tissue.
•	Elias et al. (1998) demonstrated Fos activation in neurons within the arcuate hypothalamus and retrochiasmatic area following peripheral leptin injections; these neurons were simultaneously traced retrogradely from the spinal cord (i.e., they projected to the spinal cord); this study therefore combined visualization of *connectomic* and *functional* spaces (see also Elias et al., 1999).
•	Stratford and Kelley (1999) demonstrated a functional link between the nucleus accumbens shell and the perifornical and lateral hypothalamus by showing that intra-accumbens muscimol injection triggers Fos expression in these hypothalamic regions. Similarly, Mena et al. (2013) show a functional link between the opioid-injected prefrontal cortex and the Fos-activated lateral hypothalamus.
•	Peciña and Berridge (2000, 2005) examined Fos plumes following opioid injections in the nucleus accumbens. See also Smith and Berridge (2005, 2007).
•	Hommel et al. (2006) detected phospho-STAT3 labeling following leptin injections into the ventral tegmental area.
•	Khan et al. (2007) detected phospho-ERK1 and 2 in rats given hypothalamic norepinephrine injections.
•	Williamson et al. (2010) used androgen receptor translocation to gauge the “spread” of microinjected testosterone into the medial preoptic area of rats.
•	Li et al. (2011) tracked Fos after glutamate receptor agonist injection into perifornical/lateral hypothalamus.
•	Aponte et al. (2011) and Atasoy et al. (2012) tracked Fos activation in optogenetically stimulated AgRP and POMC neurons, respectively.
**Empirical space**	
•	Demole (1927) visualized ink deposits in relation to anatomical landmarks in brain tissue cross-sections.
•	Masserman (1937) used Weil's myelin stain (1928) to visualize injection sites in relation to myelinated nerve fibers.
•	Andersson (1953), Larsson (1954), and von Euler and Holmgren (1956) visualized needles by X-rays in relation to the skull.
•	Larsson (1954) used silver staining method of Palmgren (1948) to visualize nerve fibers and nerve endings.
•	Epstein (1960b) and Grossman (1960) visualized needle scars using Nissl stains.
•	Myers (1966) evaluated the spread of various dyes of differing molecular weights to estimate diffusional distances (see also Swanson et al., 1972).
•	Booth (1968) diagrammed gliosis caused by norepinephrine injection (see his Figure 2).
•	Booth (1968), Myers et al. (1971), Leibowitz (1978), Myers and Hoch (1978), and Cole and Sawchenko (2002) tracked injectate with radiolabeled molecules.
•	Swanson et al. (1972) and Stanley et al. (1993b) developed injection delivery systems that minimize the volume (and therefore spread) of injectate delivered.
•	Scammell et al. (1996) and Cunningham et al. (2008) developed injection delivery systems that minimize damage caused by the injector itself.
•	Morton et al. (2009) injected Cy3-labeled leptin to evaluate its spread within VTA tissue.
•	Atasoy et al. (2012) detected Fos protein expression in neurons stimulated optically within the arcuate hypothalamus of mice.
•	Calu et al. (2013) correlated optogenetic silencing of dorsal medial prefrontal cortical (dmPFC) neurons with the absence of dmPFC Fos expression.
•	Thanos et al. (2013) have correlated optogenetic stimulation with altered glucose metabolism as visualized by PET.
•	Bepari et al. (2012) showed that optogenetic stimulation of striatal neurons resulted in robust mRNA expression of *Npas2, Arc*, and *Egr1* but not *c-Fos*.

*The information in this table is meant to serve as a supplement to the narrative in Section 4.4 on visualizable spaces and their use in localizing injection and probe sites in the brain. Certain seminal studies are included that may not relate directly to visualization in relation to injection or probe sites or to feeding behavior per se, but are important to note for their fundamental contributions to the visualization of the relevant space*.

#### 4.4.1. Gross anatomic space

*Gross anatomic space* represents the basic macrostructure within fixed or unfixed brain tissue, and is defined here as those features of a whole or sectioned brain that are visible to the naked eye during a gross dissection. Major landmarks, such as the cerebral ventricles, major cerebral vessels, and major white matter tracts are included in such space, and are often used to help delineate the basic location of the entry of a cannula, injection needle, microdialysis probe, or optical fiber into the brain. A good example of the effective use of gross anatomic features can be seen in the data from Eglin (1953) shown in Figure 1C, where simple drawings of sectioned cat brain indicate locations where acetylcholine and eserine produced autonomic responses.

#### 4.4.2. Angioarchitectonic space

**Figure 5:**
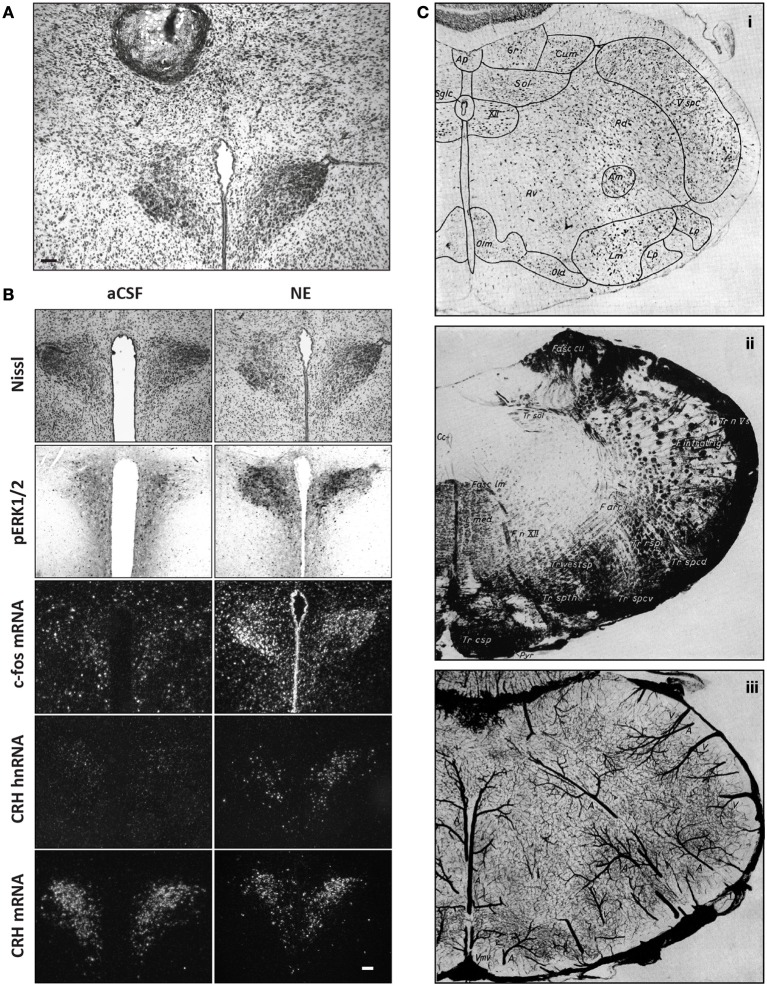
**Serial sections allow for intra-brain registration of multiple visualizable spaces**. **(A)** Nissl-counterstained tissue showing the location of an injection site in the vicinity of the hypothalamic paraventricular nucleus, as published by Khan et al. (2007). **(B)** Effects of artificial cerebrospinal fluid (aCSF) injection (left column) or norepinephrine injection (right column) on activation of paraventricular hypothalamic neurons. The first row shows the structures in cytoarchitectonic space (Nissl stain). The second row shows the structures in functional space (the label is the activation marker, phospho-ERK1/2). The third, fourth, and fifth rows show the structure in transcriptomic spaces for the molecules c-fos (mRNA), CRH (hnRNA), and CRH (mRNA). **(C)** Adjacent sections from the atlas of Wünscher et al. (1965), showing cytoarchitectonic **(i)**, myeloarchitectonic **(ii)**, and angioarchitectonic **(iii)** spaces for the same portion of the rat hindbrain. All photomicrographs in this figure were reproduced with permission from the publishers. (Panels **A** and **B**: The Society for Neuroscience; Panel **C**: Hirzel Verlag).

*Angioarchitectonic space* is defined as the space occupied by cerebral blood vessels that can be used as landmarks to help localize injections. As illustrated in Figure 5C, *angioarchitectonic space* can be visualized with other spaces in the brain.

#### 4.4.3. Myeloarchitectonic and fibrilloarchitectonic spaces

To reflect all methods used, both terms are being included here. *Myeloarchitectonic space* is occupied by myelinated axons as they traverse through brain structures, whereas *fibrilloarchitectonic space*, a term adapted here from Brodmann (1909), refers to all nerve fibers, myelinated, and unmyelinated, within the central nervous system. Although the hypothalamus is generally thought to have few myelinated fibers apart from large fiber tracts, a few investigators have stained myelin sheaths to localize injection sites in this structure. For example, recall from Section 2.2 and Figure 1H that the Leibowitz lab employed a method to simultaneously visualize cells and myelinated fibers in the same brain tissue (Table 3). Many investigators have also produced detailed studies of the myelinated components of the major fiber systems traversing the hypothalamus (Table 3). More recently, promising efforts have been made for non-invasively imaging white matter tracts in the hypothalamus using innovations of basic magnetic resonance imaging (MRI) techniques (Table 3).

#### 4.4.4. Cytoarchitectonic space

*Cytoarchitectonic space* refers to the cellular elements in the nervous system that are histologically identifiable from “elemental” or “cell type” properties (e.g., cell size and shape) or the more population-based properties for “cell groupings” (e.g., cell packing and density, regions of lamination, or clustering into larger nuclei; see Brodmann, 1909, for details). Traditional staining techniques used to determine cytoarchitecture include those developed by Golgi (1873) and Franz Nissl in the 1890's (see Nissl, 1913). In addition to stereotaxic atlases of the rat brain that include photographs of Nissl-stained material (Table 4), several other Nissl representations of the hypothalamus have been published (e.g., Bleier et al., 1979). Some investigators, however, have questioned the importance of using Nissl stains to establish cytoarchitectonic groupings of nerve cell populations (see p. 49 of Blessing, 1997). Golgi-stained material has also been generated for a number of locations in the hypothalamus (Table 3).

**Table 4:** **Reference spaces for the rat brain using coordinates in stereotaxic space**.

**Reference space^a^**	**Strain^b^**	**Sex^c^**	***N*^d^**	**Weight (s), g^e^**	**Brain^f^**	**Sections**				**Photo^i^**	**AP^j^**
						**Plane (s)^g^**	**# C^h^**	**Thickness**	**Interval**		
Krieg, 1946	(a)	ns	1	237	frs	C	29	1 mm	1 mm	N	ns
Massopust, 1956	ns	ns	30	100, 200, 300	frz	C, pS	ns	100 μm	ns	N	ns
de Groot, 1959a	(a)	ns	>1	200–300	frz/pfn	C, S	14	50 μm	400 μm	N	I
de Groot, 1959b	SD	ns	>1	200–300	frz/pfn	C, S	26	50 μm	400 μm	N	I
Morgane, 1961	SDR	mf	45	222–254	ns	C, H	ns	10–15 μm	ns	N	I (dG)
Massopust, 1961	W, OM	mf	>1	200–300	frz	C	11	50 μm	500 μm	Y	I
König and Klippel, 1963	W	f	>1	150	frz	C, S	56, 9	25, 40 μm	varies	Y	I
Szentágothai et al., 1962	ns	m	1	200	cel/pfn	C	9	ns	200 μm–1 mm	Y	β
Bernardis and Skelton, 1965	SD	m	71	50, 100, 150, 200, 250, 300	pfn	C	ns	12 μm	ns	N	I
Albe-Fessard et al., 1966	W	ns	>35	200–300	frz/pfn	C, S	30	100 μm	200–300 μm	Y	I
Fifková and Maršala, 1967	ns	ns	>1	ns	cel/frz	C	34	50 μm	500 μm	N	I
Pellegrino and Cushman, 1967	RVH	m	>1	290–320	(frz)	C (dG)	80	40 μm	200 μm	Y	I (dG), β
Szentágothai et al., 1968	ns	m	1	200	cel/pfn	C	9	ns	200 μm–1 mm	Y	β
Valenstein et al., 1969	H	mf	91	approximately 7–28	frz	C	11	80 μm	varies	N	β (KK)
Scremin, 1970	(a)	m	60	250–300	frz	C	22	300 μm	0 (continuous)	Y	λ
Sherwood and Timiras, 1970	LE	f	48	25–30; 50–55; 120–130	frz	C, S	78	50 μm	varies	Y	I
Hurt et al., 1971	(a)	mf	24	270–320	frz	C	16	50 μm	250 μm	Y	I
Ungerstedt, 1971	ns	ns	>300	ns	frz, fdr	C	19	10 μm	varies	(Y)	β (KK)
Alegría, 1973	W	m	31	175–260	ns	ns	ns	ns	ns	N	λ
Jacobowitz and Palkovits, 1974	ns	m	20	150–350	frz, fdr, pfn	C	23	14 μm	150 μm	N	I
Palkovits and Jacobowitz, 1974	ns	m	20	150–350	frz, fdr, pfn	C	22	10 μm	100 μm	N	I
Thompson, 1978	W	m	>1500	400–650	frz	C	14	90 μm	500–1500 μm	Y	λ
Heller et al., 1979	H	m	1	ns	frz	C	11	50 μm	varies	Y	β
O'Donohue et al., 1979	SD	m	ns	250–350	frz	C	18	60, 300 μm	varies	Y	I
Pellegrino et al., 1979	RVH/LE	m	>1	280–320	frz	C, S	99	40 μm	200 μm	Y	I (dG)
Paxinos et al., 1980	W	m	1 (130)	250–300		H	14	25 μm	500 μm	Y	β, I
Slotnick and Hersch, 1980	LE	m	1 (14)	345–665	frz	C	31	50 μm	120–710 μm	Y	I
Simson et al., 1981	(a)	ns	ns	150	ns	H (KK)	0 (12)	varies	varies	Y	ns
Paxinos and Watson, 1982	W	m	>1	270–310	frz	C, S, H	76	40 μm	500 μm	Y	β, I
Zilles, 1985	W	ns	12	350	pfn	C, S, H	30	20 μm	500 μm	Y^*^	I (PW)
Schober, 1986	W	m	5	300–350	cel	C	13	30 μm	900–1100 μm	Y	β
Paxinos and Watson, 1986	W	m	>1	270–310	frz	C, S, H	76	40 μm	500 μm	Y	β, I
Swanson, 1992	SD	m	1	315	cel	C	73	30, 40 μm	varies	Y	β (PW)
Kruger et al., 1995	SD	m	3	320, 328, 387	cel	C, S, H	72	20 μm	300 μm	Y	β, I
Toga et al., 1995	SD	m	1 (6)	270–320	frz	C	250–300	100 μm	0 (continuous)	(Y)	β, λ, I
Paxinos and Watson, 1997	W	m	>1	270–310	frz	C, S, H	76	40 μm	500 μm	N	β, I
Paxinos and Watson, 1998	W	m	>1	270–310	frz	C, S, H	78	40 μm	500 μm	Y	β, I
Swanson, 1999	SD	m	1	315	cel	C	73	30, 40 μm	varies	Y	β (PW)
Swanson, 2004	SD	m	1	315	cel	C	73	30, 40 μm	varies	Y	β (PW)
Paxinos and Watson, 2005	W	m	1	290 (c), 270 (s), 290 (h)	frz	C	161	40 μm	120 μm	N	β, I
Paxinos and Watson, 2007	W	m	1	290 (c), 270 (s), 290 (h)	frz	C, S, H	161	40 μm	120 μm	Y	β, I
Paxinos and Watson, 2014	W	m	1	290 (c), 270 (s), 290 (h)	frz	C, S, H	161	40 μm	120 μm	Y	β, I

This is a list of the major reference spaces, in chronological order, published for the rat in stereotaxic space. Superscripts after column headings denote the following:

a the reference space list does not include those publications which constitute non-stereotaxic atlases; thus, this is only a small portion of the total reference spaces available for mapping rat brain structures; for full citations for these reference spaces, please consult the References at the end of the article;

b for strain: (a), albino; H, Holtzman; OM, Osborn-Mendel; SD, Sprague–Dawley; W, Wistar; RVH, Royal Victoria Hospital, LE, Long–Evans;

c for sex, m, males; f, females; mf, both males and females were used;

d for sample size, or N: note that this sample number describes the total number of subjects used as listed by the authors, but does not necessarily signify that the visual spaces for the reference work were derived from all of these animals;

e *Body weights are expressed either as a range, or as a single value for the individual subject used in the reference space. In some cases, weights are specified separately for rats processed for tissue sectioned in the coronal (c), sagittal (s), and horizontal (h) planes*.

f for brain condition: fdr, freeze-dried; frs, freshly dissected; frz, frozen; pfn, paraffin-embedded; cel, celloidin-embedded; in some cases, more than one preparation was included;

g Plane(s) of section: C, coronal or transverse; H, horizontal; S, sagittal; pS, parasagittal; sometimes the plane of section is following that of another reference work, such as de Groot (dG) or König and Klippel (KK);

h #C, number of coronal sections in the reference space (note that 0 coronal and 12 horizontal sections were included in Simson et al., 1981);

i Photographs of stained tissue sections either accompanying the reference space - Y - or not included with the reference space - N - are indicated. (Y), indicates that only a few photographs were provided as a supplement to the reference space drawings;

Y^*^ *, designates, for the Zilles atlas, that photographs of stained tissue sections were used as a supplement to, but not as a basis for, the reference space drawings. The drawings, in turn, are instead based on the original histological material of Paxinos and Watson (1982)*.

j AP, anteroposterior coordinate, based on either the location of interaural line (I), Bregma coordinates (β), or Lambda coordinates (λ); in some cases the coordinates are based on another reference work, such as de Groot (dG), Konig or Klippel (KK), or Paxinos and Watson (PW). In all columns, “ns” denotes “not stated.”

#### 4.4.5. Connectomic space

Sporns et al. (2005) defined the human *connectome* as “a comprehensive structural description of the network of elements and connections forming the human brain” (p. 0245). For our purposes here, *connectomic space* is the space in the brain occupied by its large-scale network of axonal connections. These connections have been traced using a variety of methods, as summarized in Table 3. Importantly, IGI methods also involved early studies demonstrating virus-assisted tract-tracing methods to delineate feeding-related circuits in the hypothalamus (DeFalco et al., 2001), thereby marking explorations of *connectomic space* as one entry point for IGI methods to be applied to feeding control.

#### 4.4.6. Chemoarchitectonic (chemical and proteomic) space

Chemical and proteomic types of *chemoarchitectonic space* are usually “visualized” in one of three ways: inferences derived from actions of a chemical injection, microdissection of tissue, and immunocytochemistry. Strictly speaking, the first two of these methods—inferring chemical space from the actions of centrally injected chemicals or linking molecules analyzed in dissected tissue to locations in the brain—are reciprocally incomplete. In the first case, numerous studies (some of which were highlighted in Section 2) have employed ICI methods to deliver chemicals into brain regions to control food intake. These studies usually provide (a) the site of injection (i.e., location), and (b) a record of what the injected chemical did (e.g., behavior, etc.), but the identity of the endogenous chemical space affected by the injected chemical is rarely distinct. As discussed in Section 3.3 the concept of behavior as “chemically coded,” which emerged from this approach, emphasized the anatomical location of neural substrates underlying the triggering of a complex behavior.

The second method to probe chemical space, which involves microdissection and homogenization of restricted, pre-determined locations within fresh or freshly frozen brain tissue, is incomplete for just the opposite reason: the chemical information obtained from the tissue is generally accurate, but the location information provided is limited at best. Strictly speaking, this method is not “architectonic,” since the intact structure of the tissue itself is not preserved. However, microdissection atlases (e.g., Palkovits and Brownstein (1988) facilitate cataloguing of chemical space that is anchored to the physical, mapped structure of the intact tissue itself. In a general sense, then, such data constitute a form of chemoarchitecture. However, since the punches are usually quite large and encompass tissue that includes more than one brain region, it is often difficult to attribute a chemical constituent of the homogenate as being from a particular location. Sample sizes become smaller by using laser-capture microdissection techniques (Table 3).

The third (and premier form) of identification that identifies and preserves chemoarchitecture is immunocytochemical staining [reviewed by Polak and Van Noorden (1997)]. Although this method can, in principle, be used for any molecule type that is immunogenic in an animal, it has been used primarily to localize proteins in the nervous system, and hence visualize *proteomic space*. Numerous studies have used this technique to visualize feeding-related molecules, axonal pathways, cell populations, and structural and functional representations of activated feeding control circuits. These antibody-based techniques allow for intact preserved tissue sections to retain labeling for key molecules targeted by the antibodies selected. Importantly, tract-tracing experiments as well as IGI methods involving optogenetics and pharmacosynthetics can utilize antibody-based methods to localize tracers, reporter molecules, and activated neurons. This technique can be combined with counterstaining procedures to help visualize *proteomic space* simultaneously with other spaces, or with *in situ* hybridization procedures (discussed below) to co-label molecules in *proteomic* and *transcriptomic* spaces (e.g., Khan and Watts, 2004).

#### 4.4.7. Chemoarchitectonic (genomic, transcriptomic) spaces

Akin to chemical and proteomic determinations, these spaces are through which chemoarchitecture is visualized at the DNA or RNA levels. Central injection-induced activation of transcripts has been demonstrated in the hypothalamus (e.g., Khan et al., 2007; Figure 5). Within the context of feeding behavior, tract-tracing techniques to visualize *connectomic space* have been employed in combination with *in situ* hybridization methods to visualize *transcriptomic space* to determine connectivity between brain regions activated during a conditioned feeding paradigm [(Petrovich et al., 2005); reviewed by Petrovich (2011)]. Specifically, the authors injected the retrograde tracer, Fluorogold, into a portion of the lateral hypothalamic area and observed retrogradely labeled neurons in the prefrontal cortex and portions of the amygdala. Figure 1P shows the location of a representative Fluorogold injection site and its careful boundary delineations as mapped onto atlas plates from Swanson (1999). Rats traced in this manner and trained to associate food presented in their cage with an auditory cue (a tone) displayed greater levels of mRNA expression for the immediate-early genes *Arc* and *Homer1a* in the traced neurons, as compared to traced rats that did not form this association (control group). This elegant study provided the first evidence that learning associated with feeding specifically activated forebrain circuits that were connected directly to lateral hypothalamic substrates implicated in feeding control.

#### 4.4.8. Functional space

*Functional space* is defined as that space which contains elements that become visualizable depending on the behavioral or physiological state of the experimental subject. The study by Petrovich and colleagues discussed in the previous section is a great example of the combined visualization of multiple spaces in the brain: *connectomic, transcriptomic* … and *functional*. This is because the immediate early gene expression in the traced neurons provided evidence that activation in these cells occurred in association with a conditioned cue. Under our definition then, the distribution of these neurons in the brain constitutes *functional space* since the activation is associated with a behavioral change. The discussion in Sections 2.3.4.2 and 2.3.4.3 regarding the Fos plume maps of Berridge and colleagues, and the phospho-STAT3 activation following central leptin injections, respectively; are also examples of the use of activation markers to visualize functional space. Another clever instance of the adroit use of activation markers was reported by the laboratory of Victor Viau, in which they used the testosterone-dependent translocation of the androgen receptor from the cytoplasm to the nucleus as an index to gauge the extent to which testosterone delivered into the medial preoptic area had spread (Williamson et al., 2010). Figure 1R reproduces a portion of the data from their study, illustrating the punctate labeling of the androgen receptor as detected by immunocytochemistry. Table 3 provides a snapshot of a few additional studies exploring the visualization of *functional space* in the context of feeding control and other motivated behaviors. Large-scale efforts are now underway to map the functional space of the mammalian brain, as part of the Brain Activity Map project (Alivisatos et al., 2013).

#### 4.4.9. Empirical space

This space is defined as that which has been physically or functionally affected by the mechanical and chemical forces of the intracranial injection itself or the injectate that was delivered, or both. These spaces are by definition also located within the brain, but can be considered as distinctly topological. *Empirical space* therefore includes any area within the brain that is visualizable as having been affected by intracranial manipulation. Several distinct types of empirical space are discussed below.

***4.4.9.1. Damaged tissue left by a probe (e.g., cannula, needle, micropipette, fiber, light) constitutes empirical space***. In Section 1.2, we introduced several possible tissue perturbations caused by ICI and IGI methods. In the case of ICI, the delivery of a chemical solution usually causes some displacement of the tissue and the needle itself leaves a tissue scar that can be visualized, often accompanied by gliosis. If the needle was inserted through an indwelling guide cannula, the cannula itself also causes tissue damage. Investigators conducting ICI studies often localize the effects of their injections by documenting the locations of these tissue perturbations.

In the case of IGI methods such as optogenetics or pharmacosynthetics, the process of injecting solutions containing virus particles results in tissue damage similar to that found in ICI, especially if the delivery is done using blunt metal needles rather than glass micropipettes (which typically leave less visible damage; e.g., see Chamberlin et al., 1998). To ensure that a broad area of the brain receives the efficient delivery of a virus, arrays of injectors have also been used to deliver viral particles simultaneously to several adjacent regions of the same brain (Chan et al., 2010); such methods will ostensibly leave multiple injector scars. With certain IGI methods, such as optogenetics, another tissue perturbation is of the optical fiber used to stimulate or silence transduced neurons using light pulses. However, recent breakthroughs in the fabrication of microscale, inorganic light-emitting diodes (μ-ILEDs) result in intracranial optical probes leaving much less tissue damage than that left by conventional optical fibers (Kim et al., 2013b). Another consideration in optogenetics is the cellular damage that could be caused by light itself, especially if pulsed for prolonged periods of time *in vivo*. In some situations, when experimental design demands prolonged bouts of steady illumination, investigators pay special consideration to this issue by examining cellular markers of damage or inflammation (e.g., microglia or astrocytes contributing to gliosis) in the area receiving illumination (Calu et al., 2013).

***4.4.9.2. The site of injection is empirical space***. In Section 4.2, I described a few approaches by which investigators have visualized their central injection sites using ICI methods. Although many investigators have used stainless steel injector needles to deliver chemical solutions, a few investigators have opted to avoid both the greater damage and spread caused by such injectors by using glass micropipettes drawn to very small tips. A fine example of this was demonstrated by Scammell et al. (1996), who showed that 100 nl injections of prostaglandin E2 into the medial preoptic area produced fever in rats. Figure 1M shows a representative injection site from their study, which was visualized in a few ways. First the injection site was marked by injection of fluorescent beads. Second, the fluorescence image of the site was superimposed digitally onto a dark field photomicrograph of the region. Very little tissue damage accompanied the injection, and the small volumes employed helped ensure a finer degree of control over the localization of effective sites producing fever.

Knobloch et al. (2012) employed a similar strategy when they reported that optogenetic stimulation of hypothalamic-originating oxytocinergic projections to the central nucleus of the amygdala attenuates fear responses in rats. In their study, which also examined other locations of oxytocinergic innervation in the brain, the authors carefully document both the stereotaxic coordinates as well as injection volumes for their viral injectate within various brain regions, citing the nomenclature of Paxinos and Watson. Accompanying these data are also mapped locations of optical fiber placements within the central amygdala and a photograph of a glass micropipette injection site where fluorescent beads were injected at locations corresponding to where optical fibers were implanted (their Figure 5A). This is a good example of stereotaxic, diagrammatic and photomicrographic documentation of optogenetic manipulations. Finally, because neuroanatomical tract-tracing techniques involve the central microinjection of tracer molecules, *empirical space* technically can overlap with *connectomic space*. This is because the injection site where tracer deposits are found, along with all traced cell bodies and axonal connections that contain exogenous materials (tracer), also technically constitute *empirical space*.

***4.4.9.3. The area physically exposed to diffused injectate is empirical space***. Empirical space also includes that space which can be visualized for the purposes of tracking the spread of injected solutions (see discussion in Routtenberg, 1972), a task that is probably one of the most difficult challenges when using ICI methods. Recall from Section 2.3.4.1 that Stanley et al. (1993b) injected radiolabeled NPY in the small volume of 10 nl into various areas of the hypothalamus and surrounding regions, and tracked the spread of this solution in tissue by monitoring the distribution of radioactivity as a function of distance and time (Figure 3). Table 3 lists several studies that have included injections of variously labeled compounds to track diffusion of injected solutions; in all cases, differences in the molecular weights and solubilities of the tracking substance (e.g., dye) and the injected substance of interest (e.g., receptor agonist) should be taken into consideration, as well as the fact that the local concentration of drug, acting at the final receptive site *in vivo* is difficult to estimate, but will be less than the concentration at the injection site itself.

***4.4.9.4. The area functionally affected by diffused injectate: functional space as empirical space***. Finally, as discussed in the preceding section in relation to *functional space*, the activation of the underlying neural substrates affected by an injected solution can also be tracked, and the extent of this activated area is used to infer the extent to which the solution has diffused. As reviewed in Table 3, for ICI methods, these approaches have included identifying Fos plumes, phospho-ERK1/2 activation, or nuclear translocation of hormone receptors in neurons under the injected solution. As discussed in Section 3.2.3.1 for optogenetics, Fos activation following light stimulation also allows one to infer if light from the optical fiber has affected the excitability of neurons underneath the illumination, and again renders the use of *functional space* as *empirical space*.

For IGI methods, the viruses injected often carry constructs that not only encode the engineered construct to be delivered, but also a “reporter molecule,” such as a histochemically or fluorescently detectable protein (e.g., β-galactosidase; mCherry, tdtomato, EGFP). This was demonstrated early in the introduction of viral vectors as tools for gene delivery (e.g., Davidson et al., 1993; Figure 1K). When transduced within a neuron, the presence of the expressed reporter molecule signifies that the labeled cell has successfully incorporated the sequence delivered by the virus into its cellular machinery and has presumably also expressed the engineered construct as a functional protein. The distribution of the reporter molecule expression within brain tissue is therefore not only an important indicator of the total pool of neurons that are possibly receptive to optogenetic or pharmacosynthetic manipulation, but also allows one to infer the successful spread of virus to that location. Noordmans et al. (2004) adopted this approach for their overexpression of a GAD65 (glutamic acid decarboxylase) construct into the lateral hypothalamus of rats (Table 2). Their work is exemplary in that they used reporter molecule expression to estimate both the volume of the transduced area as well as the error in their stereotaxic procedure, parameters that are rarely included as documentation in IGI studies. In their optogenetic study of cardiorespiratory activity in the mouse hindbrain, Abbott et al. (2013) offer a good example of how optical fiber placements can be mapped carefully in *empirical space* and how these locations can be linked, using stereotaxic coordinates, to the distributions of reporter-expressing molecules at these locations documented in *functional* and *stereotaxic* spaces (see their Figure 2). Also, placing reporter molecule expression patterns in register with the tips of optical fibers is a useful means to establish the plausible area of the tissue that would be most likely to receive light illumination from the fiber (Calu et al., 2013). Similarly, viral-mediated RNAi methods also utilize the “reporter molecule strategy”; in such cases, cells expressing the reporter molecule mark their receipt of shRNA molecules, which then knock down expression selectively in the transduced cells (Hommel et al., 2003). Viral-mediated RNAi and overexpression have been demonstrated to affect food intake when targeted against various molecules in a number of different regions (Table 2). Collectively, these strategies effectively transform *functional space* as *empirical space*.

#### 4.4.10. Summary: visualizable spaces

At a basic, holistic level, this proposed organization of the brain into “visualizable spaces” is obvious: the brain will contain basic structures that inform its overall gross anatomy (*gross anatomic space*), including blood vessels (*angioarchitecture*), myelinated, and unmyelinated fiber tracts (*myeloarchitecture; fibrilloarchitecture*), axonal connections (*connectomic space*), cell populations (*cytoarchitecture*), including specialized chemical cell types and systems defined by the expressed, functional proteins and other small molecules, and the genes and transcripts within these cells (*chemoarchitecture*). If one's intention is to publish as much location information about a food intake manipulation *in vivo*, then data should be extracted from one or more of these visualizable spaces once the behavioral component of the study is completed. Operationally, however, it is usually the case that only one or a few of these spaces is examined histologically for any single brain specimen. That is the reason why the emphasis here is to call these methodologically separable spaces as *visualizable spaces*: the space, *although always there, is essentially absent* if it is not the space chosen by the investigator to be visualized histologically or documented diligently. As we shall see, such choices lead to profound consequences about the limits that investigators place upon their own studies.

### 4.5. Seeing visualizable spaces

“Exact information about the functional significance of the deep sections of the brain is only obtained by working through the brain histologically in serial section. To avoid far too great delays, the experiments must be fitted in together as it were in time, and it is only possible to keep the material collected under control by using a carefully organized system of registration.”

*—Walter Rudolf Hess*

*Nobel Lecture (1949)*

How can we use “visualizable spaces” to address questions about central injection sites and the neural substrates affected by such injections? The simplest case is that of histological localization of an injection site in a single brain, because this allows all visualizable spaces to be easily placed into anatomical registration with one another (*intra-brain registration*). Applying now the names of the visualizable spaces to our earlier discussion, using a Nissl stain to mark cellular populations allows one to visualize the brain in *cytoarchitectonic space*. The presence of an India ink-labeled injection site, in contrast, allows us to visualize the brain in *empirical space*. Thus, whereas Demole's ink-stained injection sites within the unstained brain in 1927 (Figure 1B) are localizable within *empirical space* and *gross anatomical space*, Epstein's Nissl-stained injection sites (Epstein, 1960b), which also contained needle tracks (Figure 1E), are localizable within *empirical space, cytoarchitectonic space*, and *gross anatomical space*. Similarly, the Klüver-Barrera-stained injection sites (Figure 1G) in Leibowitz (1975) are visualizable in *empirical, cytoarchitectonic*, and *myeloarchitectonic spaces*, and the β-galactosidase stained injection site (Figure 1K) within the unstained brain shown in Davidson et al. (1993) marks *functional, gross anatomical, and empirical spaces*. Similarly, fluorescent bead-marked injection sites in superimposed dark field images (Figure 1M) in Scammell et al. (1996) are visualizable in *empirical* and *myeloarchitectonic* spaces. In all of these cases, the tissue is being visualized simultaneously in two or three separate visualizable spaces because the methods that allow for such visualization do not interfere with each other, and all can be applied together to the same tissue section (e.g., see introductory comments in Steward, 1981). The use of these separate methods to the same tissue section is the simplest form of ensuring that there is 100% (perfect) *registration* between the visualizable spaces, *since the tissue for each space is identical*.

The issue becomes a bit more complicated if the methods to localize the injection site within two or more visualizable spaces are mutually exclusive of one another, or are technically difficult to perform simultaneously. For example, performing an immunofluorescence procedure to visualize a particular class of molecules under a fluorescent microscope may not always be fully compatible with the tissue processing steps needed to view Nissl-stained material *in the same tissue section* using bright field microscopy. In such instances, collecting adjacent series of tissue sections spaced closely together helps to solve the problem. Depending on the brain region one wishes to analyze, the degree of separation between closely adjacent tissue sections is such that the full extent of a relatively homogenous region is represented more or less equally within multiple, serially collected sections. This allows for each tissue section to be processed separately for each method one desires to use for visualizing the injection site.

Figure 5 shows this for a study in which NE or its artificial cerebrospinal fluid (aCSF) vehicle was injected near the hypothalamic paraventricular nucleus (PVH) to trigger the expression of *c-fos* and corticotropin-releasing hormone (*Crh*) genes (Khan et al., 2007). The PVH was sampled within adjacent tissue series so that at least five separate tissue-processing steps could be performed separately on the same injected region, one for each tissue series collected. This allowed for the following visualizable spaces to be examined simultaneously for the PVH in a single brain: in Figure 5A: (1) *empirical space* (injection site); in Figure 5B: (2) *cytoarchitectonic space* (Nissl series); (3–5) *transcriptomic spaces* for c-Fos mRNA, CRH mRNA, and CRH hnRNA (*in situ* hybridization series); and (6) *functional space* (induction of phosphorylated MAP kinases, pERK1/2; immunocytochemistry series). Also, Figure 20.5 of Watts and Khan (2013) shows a similar set of tissue views for other transcriptomic and proteomic spaces.

Using serially collected sections that sample one region uniformly still allows one to place the visualizable spaces from each series in registration with one another. Taking again the data in Figure 5 as an example, if one wished to examine the tissue series labeled for c-fos mRNA and ask in what brain region is the mRNA being expressed in response to an injection of NE in the region, one merely has to examine the adjacent Nissl series (panel **B**, top), and conclude that the c-Fos mRNA is in a region that is co-spatial with the region in the Nissl series that stains as a densely packed collection of cells corresponding to the paraventricular hypothalamus. Thus, to draw a conclusion about the *cytoarchitectonic space* for a tissue section containing only mRNA, one can “consult” adjacent *cytoarchitectonic space* containing the same brain region. Figure 5C illustrates how neuroanatomical reference works often include multiple serial tissue series for a region, each processed for separate visualizable spaces. In the case illustrated, which is from a beautiful atlas of the rat hindbrain by Wünscher et al. (1965), the hindbrain regions that contain integration structures relevant for feeding and metabolic control, such as the area postrema and nucleus of the solitary tract, can be visualized in the context of *cytoarchitectonic* (Figure 5Ci), *myeloarchitectonic* (Figure 5Cii), and *angioarchitectonic* (Figure 5Ciii) spaces. Similarly, the *chemoarchitectonic* rat brain atlas of Paxinos et al. (2009) includes stained sections for seven cellular markers, each from a separate series from a single brain.

#### 4.5.1. Visualizable spaces within the intact, unsectioned brain: clarity as a solution to the intra-brain registration problem

Recently, the Deisseroth laboratory at Stanford University has reported the development of a tissue clearing method called CLARITY, which allows for the selective removal of lipids from the intact brain and their replacement by a nanoporous hydrogel that cross-links effectively with proteins, nucleic acids, and small molecules within cells; thereby preserving the overall cellular structure of the brain as a “tissue-hydrogel hybrid” (Chung and Deisseroth, 2013; Chung et al., 2013). Although the announcement of this new technology is very recent at the time of this writing, evaluation of the initial reports of its development suggest that the approach has the potential to greatly simplify the problem of having limited visualizable spaces within the brain that can be examined simultaneously. For one thing, tissue from a brain treated by CLARITY does not require sectioning; therefore, there are no sections that have to be placed in registration with one another. However, this is only one half of the challenge; the other would be to ensure that the brain, if not sectioned into adjacent series, would still be visualizable with multiple labels that would be chemically compatible with one another. This half of the challenge, too, seems surmountable with CLARITY, as the technique allows multiple rounds of immunocytochemistry, for example, to label the same brain structures through the use of electrophoretic tissue clearing (ETC) (Chung et al., 2013). In principle, the labeled (and transparent) brain could be imaged before each new round of labeling, so that the images could be superimposed upon each other in the same holistic visualizable space (which perhaps should be called “*clarified space*”). *Intra-brain registration* issues, therefore, appear soluble using this technique; however, as introduced in the next section, the challenge of *inter-brain registration* may well remain for some time even for this exciting new technology. Indeed, Chung et al. note (p. 510): “Turning immense data sets into useful insights remains a key challenge. Computational approaches to image segmentation, three-dimensional registration and automated tracing require further development.”

### 4.6. Registration of visualizable spaces within two or more separate brains

“A concept (e.g., of a neuron) is the fruit of one's investigative approach to an object of nature. If one's approach had led to knowing the object, one would possess the truth with respect to it. It is for me an apriorism that so successful an approach is non-existent: truth is not to be had. The most we attain is an image of truth: a concept.”

*—Hendrik van der Loos (1967)*

*“The History of the Neuron”*

I open this section with the insight of van der Loos, because the registration of visualizable spaces becomes most powerful if it can be achieved with two or more *separate* brains; that is, if the visualized space in one brain can be “snapped into place” with a different space in another brain. The benefits of such a marriage are obvious: data across multiple studies, generated by separate labs in separate lands, could, in principle, be interrelated together. Inter-brain registration is a holy grail in neuroanatomy, the attempted solutions for which have involved complex registration efforts (e.g., Zitová and Flusser, 2003; Zaslavsky et al., 2010). Yet, even if we were to somehow place into registration, say, spatial information about an injection site from one brain with the spatial information provided by neuroanatomically traced neurons corresponding to that location in another brain, the most we attain is, as van der Loos states, *an image of truth: a concept*. This is to emphasize that there is no substitute for empirically determining your injection site or probe location in relation to the structures within the *same* brain. Anything short of this starts one upon a path of determining placement locations with ever-increasing sources of error, an idea that will become clearer as we explore ways to interrelate the visualizable spaces of *different* brains. As there are two possible scenarios for *inter-brain registration* of visualizable spaces, each with its own challenges, each will be dealt in turn.

#### 4.6.1. Reference space as a solution to the first scenario

**Figure 6:**
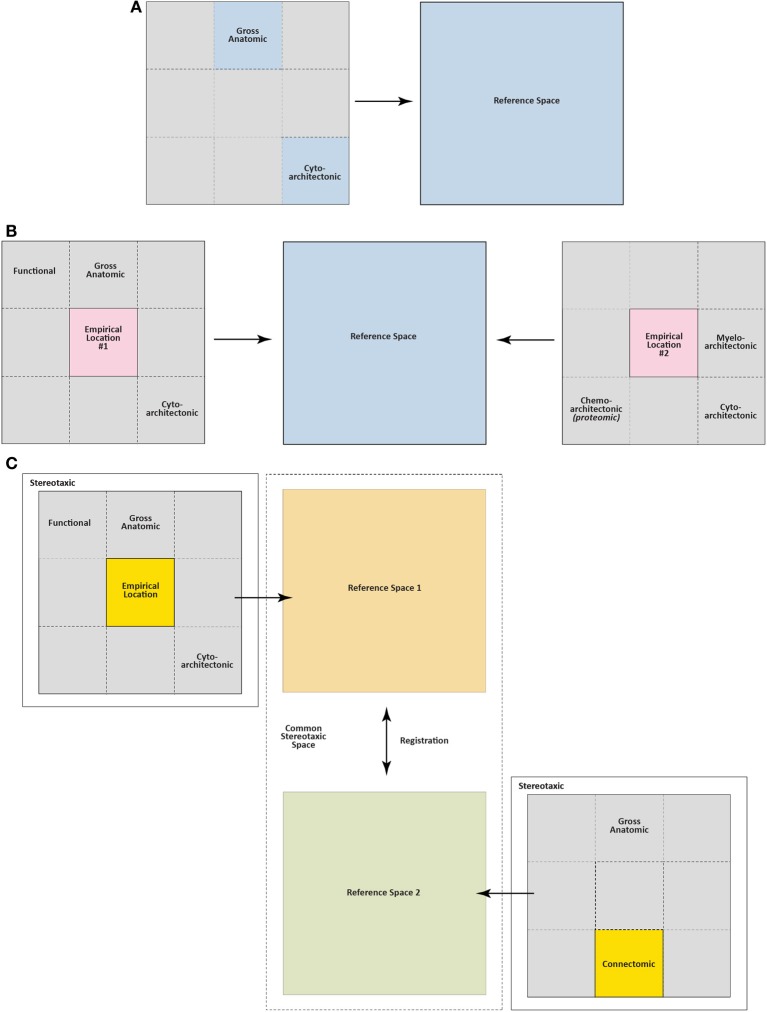
**Reference space, stereotaxic space, and anatomical registration**. **(A)** A reference space derived from the spatial analysis of a rodent brain. In this case, the visualizable spaces used to create the reference space include *gross anatomic space* and *cytoarchitectonic space*. **(B)** “Scenario One” for inter-brain registration, as described in Reference space as a solution to the first scenario. Mapping of two empirical locations (injection sites, for example) from two different brains to a common reference space. **(C)** “Scenario Two” for inter-brain registration, as described in Stereotaxic space as a coarse but viable solution to the second scenario. Datasets from two different visualizable spaces (*empirical* and *connectomic*) and from two different brains have each already been mapped to two different reference spaces, respectively. To bring these into registration (at least along a rostrocaudal axis) requires that they share common stereotaxic coordinates.

In the first scenario, two visualizable spaces in two separate brains appear to be in close correspondence with one another but it is difficult to verify this precisely. For example, the injection sites from one study may be in similar locations to those in another study, but differences in the plane of section of the tissue, or how the tissue was visualized, or how the visualizable space was *documented* in the study, or all three factors; may prevent precise knowledge of this fact. The solution to this scenario, as with many others like it, is to experimentally map the locations of the injection sites from one experiment to a *reference space* (Figure 6A), and to use the *same* reference space to map the injection sites of the second study. Thus, the relation becomes 1:*R*:2, where the visualizable space in one brain (1) and another (2) are mapped to a common reference space (*R*), as shown in Figure 6B. We have already dealt with an example of this in the first part of this article: recall in Figure 2E that the crystalline deposit sites for NE mapped by Grossman (1962) were in the reference space of de Groot's atlas, and the same reference space was used by Booth (1967) (Figure 2F) to map locations where he had injected the same neurotransmitter. The de Groot atlas, therefore, serves as one *reference space* that permits juxtaposing two datasets generated five years apart and in two separate laboratories.

The beauty of using a reference space is that it links datasets in space and time, and the number of possible datasets such a space can hold is theoretically limitless, especially if the reference space exists in a digitally extensible format. Table 4 provides a list of reference spaces, for the rat brain or structures within it, that take advantage of *stereotaxic coordinate space*, which will be discussed shortly. Many of the more recently published reference spaces listed in the table have vector graphics files, such as atlas templates, in digital formats to accompany them. These digital atlas templates are incredibly useful for mapping experimental data in a reproducible and efficient manner. Although we have focused on the rat in this discussion, there are many other reference atlases available that can and are aiding behavioral studies. Some, such as the Allen Brain Atlas for the mouse (Lein et al., 2007), are searchable on the web (http://mouse.brain-map.org/), accompanied by a reference atlas in print form (Dong, 2008), and are being used as a springboard for new resources for facilitating IGI studies (e.g., Madisen et al., 2010). High-resolution atlases of the mouse brain (Li et al., 2010) and human brain (Amunts et al., 2013) have been published. Reference brain atlases for invertebrates also have been created, such as those for the *C. elegans* nematode (White et al., 1986; Hall and Russell, 1991), *Heliothis*, and *Manduca* moths (Kvello et al., 2009; Huetteroth et al., 2010), *Tribolium* red flour beetle (Dreyer et al., 2010), locust (el Jundi et al., 2010), honeybee (Rybak et al., 2010), and *Drosophila* fruit fly (Chiang et al., 2011; Shinomiya et al., 2011; Milyaev et al., 2012).

Of course, there are caveats with “jumping into” a reference space: expert knowledge is required to accurately “map” experimental data to such a space. It is important to remember that a reference space is originally created from the histological examination of a brain or set of brains in a few key visualizable spaces (Figure 6A). Therefore, your own experimental data, within the brain specimen you have processed, is being compared to another brain or set of brains. Just as you make choices about which visualizable spaces to detect in your tissue sample (e.g., “Should I use a Nissl stain, Weil stain or *in situ* hybridization on this tissue series?”), so too, have the authors in Table 4 made choices as to how to represent their reference spaces, and on what basis to create such spaces. For example, as shown in Figure 5C, for the atlas of Wünscher et al. (1965), the authors decided to utilize three visualizable spaces: *cytoarchitectonic* (Figure 5Ci), *myeloarchitectonic* (Figure 5Cii), and *angioarchitectonic* (Figure 5Ciii).

How does one accurately map experimental data to a reference space? Extensive discussion of this intricate issue is outside the scope of this article, but readers can turn to Swanson (2001, 2004), and Simmons and Swanson (2009); for one viewpoint concerning this topic. Essentially, experimentalists must choose structures within the visualizable spaces they can view, and utilize these chosen structures as *fiducials* with which to map their data onto a reference atlas. Of course, it is highly preferred to plan ahead and section the material from the completed experiment as close as possible to the plane of section used by the reference atlas chosen. The preferred fiducial structures to choose in one's sample are those basic landmarks that are included in the reference space as well, which will allow one to match one set of structures to the other. In making such selections, it is helpful to remember that it less likely for structures along the midline of the brain to vary drastically in the left vs. right hemispheres if a transverse section is sectioned with the intention of keeping both hemispheres similar in appearance to one another. Thus, “stable” structures such as the anterior commissure, or midline hypothalamic structures such as the suprachiasmatic nucleus, paraventricular hypothalamus, and rostral pole of the arcuate hypothalamus; are good fiducial structures that can be matched to the corresponding structures in a reference atlas. Comparing both sets of structures allows one to determine how close or far in plane the experimental tissue has been sectioned from that of the brain sectioned for use in the selected reference atlas. Although mapping experimental data to a reference space is not always straightforward, the rewards of careful effort are that investigators can now interrelate their data with any other data mapped to the same reference space.

One final note deserves mention about the use of reference spaces to map probe locations in the brain. Depending on how the brain used to produce the reference space is sectioned, a certain interval will exist between sections, which usually can vary for that space depending on which portion of the brain is sampled. For example, many atlases typically sample only a small portion of the olfactory bulb in the rat brain, and show therefore only a few representative plates from this generally longitudinally invariant structure. In other parts of the brain, the sampling frequency may be higher. Because of this, situations will arise when the experimenter finds the probe location they wish to map to fall exactly *between* reference plates in an atlas. In such situations, a choice is made to either map the location of the injection to the nearest atlas plate, or to forego mapping the location for that particular case. In rare instances, the authors will have access to the original tissue sections that form the basis of the reference atlas and find the unpublished section from the original series that falls between the mapped sections, and have a new map drawn for it (Kelly and Watts, 1998; Watts and Sanchez-Watts, 2007). To date, this author is not aware of chemical injection studies that have explicitly indicated on their maps whether they include injection site locations falling between reference atlas intervals. It is interesting that in his systematic exploration of electrical stimulation sites within the brain, Hess would distinguish sites “fitted” to the nearest reference plates from those “in register” with them through the use of distinguishable symbols marked on the summary maps themselves [e.g., see pp. 30–31 and pp. 64–65 of Hess (1957) for a description and representative maps, respectively, for his symbols]. Perhaps this practice should be revived.

#### 4.6.2. Stereotaxic space as a coarse but viable solution to the second scenario

In the second scenario, two visualizable spaces in two separate brains have been mapped to two separate reference spaces, and the experimentalist does not have access to the original tissue sections for either brain, just the maps within the two separate reference spaces. Thus, the relations are, 1:*R*_1_ and 2:*R*_2_—where 1 and 2 are visualizable spaces from two separate brains and *R*_1_ and *R*_2_ are two different reference spaces. How does one reconcile these separate datasets? In this case, something must tether one set of data to the other. Fortunately, if both reference spaces contain information from *stereotaxic coordinate space*, such information could provide the tether needed to link the datasets (Figure 6C), provided that certain conditions are met.

***4.6.2.1. Stereotaxic space***. This is the only visualizable space discussed so far that is not residing in the brain *per se*, but is usually considered *before* intracranial manipulations are made in the live animal, when a *stereotaxic atlas* is consulted. Notice in Figure 4C its privileged position as a “visualizable space” surrounding the brain's other visualizable spaces, in relation to the complementary position held topologically by the skull (Figures 4A,B). Table 4 provides a list of stereotaxic atlases or individual stereotaxic studies for the rat. *Stereotaxic space* is three-dimensional space that is calibrated using skull reference points that, for the most part, do not change drastically so long as subjects fixed within the stereotaxic frame fall within a body weight range similar to that for the subjects used in the stereotaxic atlas (e.g., Paxinos et al., 1985), and the atlas ‘horizontal plane’ is used to place the subject in a similar orientation using the incisor bar of the stereotaxic instrument.

*4.6.2.1.1. True vs. inferred stereotaxic space*. Before an experiment, stereotaxic space is “visualizable” insofar as the movement of the stereotaxically held probe is concerned during the course of an experimental manipulation. This is when the coordinates are used to move glass pipettes or other delivery devices to deposit tracers, toxins, viruses, or chemical solutions (or implant chronic cannulas, microdialysis fibers, carbon fibers, electrodes, or optrodes, etc.) into pre-determined target locations based on Cartesian coordinates calibrated from surface features of the skull, which serve as “zero” points of origin. I refer to this as *true stereotaxic space*. After an experiment, stereotaxic space remains useful for estimating the position of histological material as it may have appeared in the original coordinate space, thereby allowing visualizable material to be mapped to a stereotaxic atlas. Of course, other criteria are needed to map such material, but the *post-hoc* invocation of an estimated coordinate in the anteroposterior axis, for example, is useful for basic registration purposes. I refer to this as *inferred stereotaxic space* (i.e., that which was not truly measured using a stereotaxic instrument firsthand).

True stereotaxic space may still play a role after an experiment under a few conditions. First, positional needles can be inserted into the brain, while it is still in the skull and the skull is in the stereotaxic instrument. The needles can be inserted in various orientations, and if their complete tracks appear during tissue sectioning, the experimenter knows that the tissue is being cut in register with the needle's original orientation (e.g., Paxinos and Watson, 1986). Thus, a full-length needle track appearing on the face of the tissue block marks the correct plane of section. Although the tissue is being processed after the experiment, it retains a mark of something positioned in true stereotaxic space. Second, brains can first be sectioned in stereotaxic space by keeping the skull in the stereotaxic instrument, chipping away the dorsal surface of the skull to expose the brain, and then using a blade fitted within the stereotaxic manipulator arm to produce large slabs of the brain at fixed intervals. For example, Burke et al. (2009) have used this method for the brain of a non-human primate. A slab could be cut further on a microtome to produce thinner sections that are parallel to its cut surface (Ireland and MacLeod, 1993).

**Figure 7:**
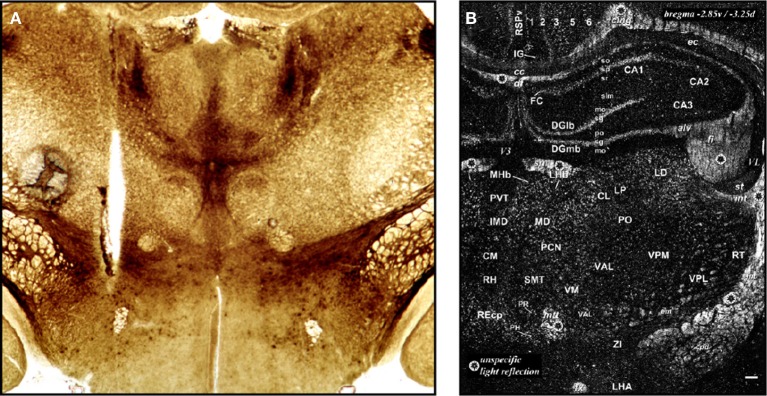
**True vs. inferred stereotaxic space**. In **(A)**, which is a coronal section from a plate in Paxinos and Watson (1986), an example of tissue visualized using true stereotaxic space is shown. The needle track marks the location of a needle traversing the section through its cut plane while the subject was placed in a stereotaxic frame. **(B)** In contrast, stereotaxic location is only inferred in the histochemical labeling of the NPY Y1 receptor by Kopp et al. (2002). Note in the upper right corner the coordinates listed in relation to the dorsal or ventral aspects of the tissue section. Both panels reproduced with permission from Elsevier.

Figure 7 illustrates tissue sections that exemplify both outcomes of stereotaxy: true and inferred. In Figure 7A, a portion of Plate 31 from the 1998 Paxinos and Watson atlas is shown, which has been sectioned “in plane” to the remaining atlas as seen from the needle track in the tissue that runs more or less continuously through its dorsoventral aspect on one side. Plate 31 was sampled at a Bregma coordinate of −2.80 mm; that is, 2.80 mm behind the Bregma suture on the skull. In contrast, Figure 7B shows a portion of immunohistochemical labeling for the Y1 subtype of the NPY receptor family in rat brain tissue, which was published by the Hökfelt laboratory (Kopp et al., 2002). The image was photographed under dark field illumination and the bright white labeling marks the immunoreactivity. Notice that in the upper right corner of this figure is a pair of coordinates: “−2.85v/−3.25d”; which indicate that the lower portion of the section is estimated to be in a transverse plane located 2.85 mm posterior to Bregma, whereas the dorsal portion of the section would be in a plane 3.25 mm posterior to Bregma. Clearly, the section was not cut *a priori* to align itself uniformly with any one level of a reference space, but the authors were careful in not only trying to helpfully provide inferred stereotaxic space information for this section, but to distinguish between differences in plane between the upper and lower parts of the section. This valuable information allows a reader of their report to independently align this labeling pattern to a stereotaxic reference space of their choosing. For example, consulting these inferred coordinates against the rat brain atlas of Swanson (2004), one can conclude that this tissue section falls somewhere within levels 28 and 29 of that atlas.

The distinction between true and inferred stereotaxic space is not simply semantic. The use of the word “true” for one space is not meant to indicate the other as “false”; indeed, both are equally useful depending on the situation. Moreover, both are just as easily prone to be inaccurate because of methodological error. The major differences, however, are the sources of the error for each type of space measure. For true stereotaxic space, incorrect probe or needle movements in any one of the three dimensions will throw a coordinate off its true mark. Many factors can make the final probe location vary from the intended location. For example, differences between the size and age of the animal used in one's study relative to that used for the reference atlas being consulted can introduce errors in stereotaxic placements. In the case of inferred space, the within-tissue patterns of labeling and fiducials that one uses to infer positional information is a matter of correct interpretation; incorrect judgments can introduce error to the estimate.

Thus, true stereotaxic space is space that is being navigated *before and during* the journey, when the ship's captain is “plotting a course” and “holding course”: in such cases, poor navigation and a failure at course correction will render the ship off course. In contrast, inferred stereotaxic space is unnavigated space that is being assessed *after* the journey has been made: in these cases, a failure to estimate one's position based on landmarks at the destination (azimuth, compass heading, coastlines) will result in a grossly overestimated or underestimated final position. Examine both panels of Figure 7 closely: from the tissue alone (ignoring labels and needle track), one does not necessarily know if (a) the stereotaxic space was measured; or (b) if it was measured, whether it is true or inferred. Generally speaking, details about the methods must provide that information (e.g., see Noordmans et al., 2004; discussed in Section 4.4.9.4). If they do not, the data are not so easily interpretable with respect to stereotaxic space. When interpreting an injection site location, it is critical to know how the location was measured, so as to be aware of the types and sources of error for the measurement.

***4.6.2.2. Stereotaxic coordinates in publications protect location data***. Information about stereotaxic coordinate space is critical to document alongside any dataset for which a precise localization of a probe, injection, or field of affected cells is needed, especially if the atlas used in conjunction with the coordinates falls out of common use over time, or if data from other types of visualizable space are not included in a publication containing the dataset. This is because the stereotaxic measurements are with respect to skull surface landmarks that are *independent* of the atlas being used. This fact has enormous implications: the proper documentation of coordinates within a publication prevent the anatomical data of an injection study from become un-interpretable by allowing for the locations of the injection sites to potentially be placed in register with a more current stereotaxic atlas. Since the newer atlas also is calibrated with respect to skull landmark position, the inclusion of coordinates for *stereotaxic space* within the older study could potentially liberate its data, preventing them from languishing in a *reference space* that few people use any longer.

***4.6.2.3. Atlas registration***. Returning, then to the second scenario discussed earlier: 1:*R*_1_ and 2:*R*_2_; two separate visualizable spaces, mapped in two separate reference spaces, can be placed in registration with one another by using stereotaxic coordinates that both reference spaces can relate to. However, this is possible only if certain conditions are met. For example, if *R*_1_ and *R*_2_ both have atlas plates that are calibrated to Bregma (the reference point on the skull surface where the coronal and sagittal sutures intersect), then they are potentially registerable with one another. Another critical factor is whether the basic angle of the surface of the skull with respect to the horizontal plane of reference used by each reference space is similar for both *R*_1_ and *R*_2_.

**Figure 8:**
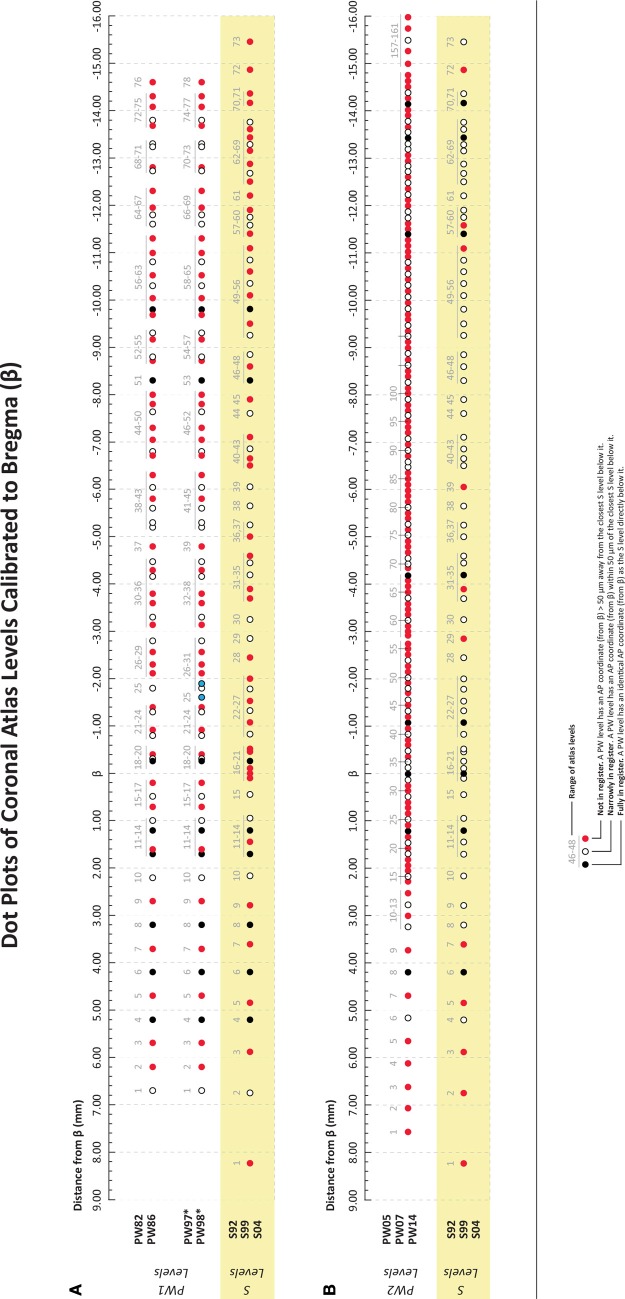
**A reference chart to align ten canonical rat brain atlases along a uni-dimensional coordinate**. **(A)** PW1 Levels = Atlas Levels from Paxinos and Watson Reference Space 1. This reference space consists of atlas levels from the first four editions of *The Rat Brain in Stereotaxic Coordinates* by George Paxinos and Charles Watson, published in 1982, 1986, 1997, and 1998 (designated PW82, PW86, PW97, and PW98), respectively. Editions 2–4 contain refinements of the atlas drawings in the first edition, but the actual tissue sections on which the drawings are based are the same as those used in the original edition. ^*^The only exception to this rule is that PW97 and PW98 differ from PW82/86 in the addition of two levels from the original tissue set that had not been published in the earlier editions. These are highlighted as blue-colored dots. Because these additions alter the numbering scheme for the PW97 and PW98 levels from those of previous PW editions, they have been displayed separately from those editions. **(B)** PW2 Levels = Atlas Levels from Paxinos and Watson Reference Space 2. This second reference space for Paxinos and Watson consists of atlas levels from the fifth, sixth, and seventh editions of The Rat Brain in Stereotaxic Coordinates by George Paxinos and Charles Watson, published in 2005, 2007, and 2014 (designated PW05, PW07, PW14), respectively. They are in a separate reference space because the tissue used was from a different animal than that used for the earlier editions, which were actually based on tissue sections from several animals. For both panels **(A,B)**: S Levels = Atlas Levels from Swanson Reference Space. This reference space for Swanson consists of atlas levels from all three editions of *Brain Maps: The Structure of the Rat Brain* by Larry W. Swanson, published in 1992, 1999, and 2004 (designated S92, S99, and S04), respectively. They are all within one reference space because the same tissue set has been used for each edition, with the editions differing primarily in the refinement of the drawings from this single tissue set. The atlas levels in PW1 or PW2 can be aligned with those in S space along the anteroposterior axis using a millimeter scale based on the skull suture landmark, Bregma. Atlas levels from PW1 or PW2 space that are in 100% registration along this dimension with a corresponding level of S space shown directly below it are depicted by a black dot (signifying “fully in register”). Those levels of PW1 or PW2 space that are within 50 μm of corresponding levels in S space are marked as white dots (with black borders). These levels are “narrowly in register” with one another. Finally, those levels between PW1 or PW2 spaces and S space that are greater than 50 μm apart are marked by red dots as “not in register.” See text for details.

Figure 8 presents a system for registering two families of atlases for the rat brain that have been in popular use: those published by Paxinos and Watson (1982, 1986, 1997, 1998, 2005, 2007, 2014) and Swanson (1992, 1999, 2004), hereafter referred to as *PW* and *S*, respectively. For the following descriptions, the reader is asked to refer to the left side of Figure 8. The PW atlases actually fall into two subgroups: *PW1* refers to the first four editions of the atlas (1982, 1986, 1997, and 1998; PW82, PW86, PW97, PW98, respectively) all of which contain the same histological dataset (i.e., they all use the same brain tissue from the same subjects). Each new edition in the *PW1* grouping is an improvement upon the previous edition in important ways: PW86 and PW97 contain a revised nomenclature over PW82, PW97 and PW98 include two additional atlas plates that were not included in PW82 and PW86. In contrast, PW05, PW07 and PW14, which form *PW2* reference space, are based on a completely different rat brain, one processed to create the atlases anew.

The upper scale in Figure 8 is in millimeters rostral or caudal to the skull suture landmark, Bregma. Two sets of comparisons are made. In the upper panel, *PW1* reference space is compared to *S* reference space, with atlas levels for these spaces marked by dots aligned along the Bregma scale to the nearest hundredths of a millimeter. Thus, PW82 and PW86, which share the same numerical scheme for atlas level numbering, are shown along the top row, with identical dots for their levels. The inclusion of two extra plates in PW97 and PW98 shifts the atlas level numbering scheme by two, thereby requiring a separate row of dots alongside PW82 and PW86 (with the new plate additions noted by blue dots). Finally the lowest row in the upper panel is for S92, S99, and S04 atlases of *S* space, all of which are from one brain and share the same numerical atlas level designations. In the lower panel, the same system has been constructed for *PW2* reference space in comparison to *S* space (which is reproduced from the upper panel).

The final set of information that allows for the reader to interpret the chart in Figure 8 is about the dot symbols themselves. One reference space's levels (*colored dots*) need to be seen in relation to the other reference space's levels *above or below it*. Red dots signify atlas plates between *PW* and *S* spaces that are *not in register* with one another with respect to the Bregma coordinate. Black dots, in contrast, represent atlas levels in each space that are *fully in register* with one another by Bregma coordinate, out to the hundredths of a millimeter. Finally, white dots denote atlas levels within 50 μm of one another, i.e., *narrowly in register*.

A few observations can be made about these alignments. First, the outer diameter of a 33-gauge needle (typically among the smallest used for ICI methods) is 120 μm. Optical fibers for use in rats are typically between 100–500 μm in diameter (Zhang et al., 2010). Thus, the resolution of the atlas level registration conducted here between the reference spaces is well within the resolution of a single optical fiber or cannula needle, or recently developed μ-iLED-based semiconductor probes (with integrated assemblies being 200–400 mm wide; Kim et al., 2013b). The resolution is also far greater than the diameters of optical imaging probes (~350 μm) being developed for deep, direct visualization of hypothalamic and other structures (Kim et al., 2013a), and about the same order of magnitude as the diameter of a glass micropipette delivering virus or tracer (typically 10–50 μm).

Second, although details of this are being readied for publication elsewhere, key fiducials (tissue landmarks) within atlas photomicrographs of Nissl-stained sections corresponding to levels marked by “black” dots, which represent levels between the two atlas systems that are in full register with one another, appear to match up very well. Thus, two sets of structural information, one dependent on the planes of section and nearest neighbor relations within the tissue (fiducials), and the other independent of the brains (stereotaxic coordinates along Bregma), are consistent with one another and serve as a validation of this arrangement (Wells and Khan, 2013). Third, notice how levels in *PW2* space are much more closely spaced than those in *PW1* space: this is because tissue was sampled every 120 μm from the brain used to create the atlas in *PW2* space; therefore, there is slightly more than twice the sampling frequency for *PW2* over *PW1* space. A consequence of this is that the ability to register levels of *S* space changes with respect to the two *PW* spaces: notice how more *S* levels are red (“not in register”) alongside *PW1* levels, than when they are alongside *PW2* levels. The greater sampling frequency of *PW2* space allows for greater amounts of data between the two atlases to be in register with one another, underscoring the interdependency of different reference systems as they evolve and improve over time.

A major limitation of this atlas interconversion system is that it is only valid along one dimension of the three-dimensional Cartesian system that represents stereotaxic space. This is a critical point. Indeed, the mediolateral and dorsoventral dimensions between the two brains are very different, since the brains were histologically processed in different ways for each reference space (Wells and Khan, 2013). In *PW* atlases, the brains were frozen and then sectioned; in *S* space the brain was embedded in celloidin and therefore underwent a shrinkage that requires correction factors in both of these dimensions (the reader is referred to the front matter of the atlases for details about these processing steps). As noted by Swanson (2001), *S* space was actually aligned and linearly rescaled to be placed in register with *PW* space in the anteroposterior dimension; it is this rescaling that allowed stereotaxic coordinates to be inferred for *S* space [see pp. 173–174 of Swanson (2001)]. Thus, *PW* space is *true stereotaxic space*, and *S* space is *inferred stereotaxic space* based on alignments and linear re-scaling of *S* space to *PW* space. This alignment and rescaling is possible because the two reference spaces are based on brains cut in very similar planes of section, using the brains of relatively similar sized rats (albeit different strains). Importantly, this conversion system allows for only a somewhat coarse atlas registration along the rostrocaudal coordinate for studies that have probe placements in the dimensions specified above. True reference space inter-registration requires computational power, a topic that has received much attention in the field of brain imaging (e.g., see Toga and Thompson, 2001).

### 4.7. To map or not to map?

“The constraint of organized life offers a better approach to truth than does an uncontrolled imagination.”

*— Jean-Paul Schaer (1987)*

*The Anatomy of Mountain Ranges*

There are perfectly defensible reasons why atlas-based approaches in neuroanatomy are not necessary for gaining an understanding of the location of probe placements or elements forming neural circuits underlying behavioral control. Armed with a Nissl-stained tissue series and a simple *camera lucida* set-up, one can describe with great accuracy where one's placements might be located in the brain and how these placements are related to surrounding populations of neurons. In some ways, representing histology through *camera lucida* drawings or photomicrographs of the stained tissue itself more accurately represents such data than a “reference map” would. However, as we shall see, a limitation of this approach is that it is very difficult to participate within a community-based effort to understand complex neural circuitry without placing one's dataset within a reference space that allows for inter-brain registration of data and for sophisticated databasing and computational tools to be brought to bear on the shared datasets. To be sure, mapping raw data to a reference space is an act of transformation that necessarily renders them into a form that is constrained by the interpretations inherent in the parcellation schemes of the reference space itself. But so long as the scientific community understands that a map of such data in reference space constitutes a *data-constrained model* of how circuits controlling behavior might be organized, which leads to one being able to test the model and refine it, then the approach has great merit. Of course, a *conceptual model* can be formulated without mapping to a reference atlas, but the model is based solely on the accumulated informal understanding by scientists of the locations where behavioral effects occur across multiple studies, without actually having the data from those studies integrated together formally (i.e., conventional wisdom). One therefore can easily suppose that a traced circuit in one study might be involved in feeding control, based on examining the injection sites in another study. Atlas-based data integration constrains and tests such suppositions so that a *shared conceptual model* becomes a *shared data-constrained model*, thereby allowing what is known about the organization of the brain itself, as Schaer notes above, to limit the “uncontrolled imagination” about how such organization might look like. In the next section, I discuss how data integration could allow for new insights to form and new hypotheses to be constructed that are constrained carefully by the shared information provided by accurately juxtaposed datasets.

## 5. Proposal: toward a community-based understanding of feeding control circuits

In 1854, the English physician John Snow made history in the fields of epidemiology and medical geography by superimposing two seemingly unrelated location datasets of the Soho district of London upon one another. The first dataset was on a map and consisted of locations of the water pumps distributed throughout the neighborhood. The second dataset was his tally, by street, of the number of deaths in each household within that neighborhood, brought about as a result of a devastating cholera outbreak. While the mode of transmission for cholera was still believed to be airborne, Snow had long felt that the illness was transmitted through water: the superimposed locations of the water pumps and deaths served to illustrate this convincingly. He published his “spot map” (Dean, 1976) to illustrate his prior conclusions, providing visual evidence that the source of the outbreak was the Broad Street water pump (Brody et al., 2000; Johnson, 2006). The lesson here is that superimposing different datasets onto a common reference space can help communicate important insights about biology, or in Snow's case, pathobiology.

In the preceding sections, I have made the case for the close examination of neuroanatomical data within central microinjection studies. I first reviewed this field in Sections 2 and 3 highlighted ways to contextualize these studies within a theoretical framework of “*visualizable spaces*” in Section 4, and also discussed in Section 4 how *intra-brain* and *inter-brain* registration of datasets can be accomplished to a first approximation. Here, I illustrate the payoffs of adopting such approaches, with a worked example of how spatial information within diverse visualizable spaces can be juxtaposed to powerfully inform us about feeding control substrates and circuits. As we shall see, the field of ingestive behavior research is potentially poised to act in a manner analogous to Snow in superimposing various datasets upon one another, with the hopes of gaining new insights about feeding control circuits in the process.

### 5.1. Example: NPY feeding circuits

Recall from Section 2.3.4.1 that the feeding effects of NPY were examined systematically in a cannula-mapping study performed by Stanley et al. (1993b). In that study, they identified the perifornical hypothalamus as a site most sensitive to the orexigenic effects of centrally *nanoinjected* NPY. They also identified several other regions in the vicinity of the perifornical hypothalamus that also contained neural substrates sensitive to the peptide. Importantly, Stanley et al. also tracked the spread of radiolabeled NPY within tissue and were able to plot its recovery as a function of distance and time traveled from the injection site rostrocaudally. Finally, the locations of their effective injection sites were plotted within a reference space, the stereotaxic rat brain atlas of Paxinos and Watson (1986), which in the system of atlas registration presented in Figure 8, is part of *PW1* reference space.

In the context of the topics discussed in Section 4 concerning visualizable spaces, atlas registration and atlas-based mapping, a few important facts present themselves concerning the NPY data just described: (1) the spread of the injected solution was empirically determined through defined distances in tissue; (2) certain atlas plates in *PW1* reference space, containing NPY-sensitive sites, are partly or fully in register with levels of *S* reference space; (3) anterograde tracers such as PHA-L have been deposited at rostrocaudal levels that are in the vicinity of the NPY injection sites, thereby delineating neural efferent pathways from those regions; (4) these traced cells have been mapped in *S* reference space; and (5) the feeding-sensitive sites are situated near the known distributions of key feeding-related cell populations and receptors.

#### 5.1.1. Aligning empirical, stereotaxic, and reference spaces to evaluate NPY feeding circuits

**Figure 9:**
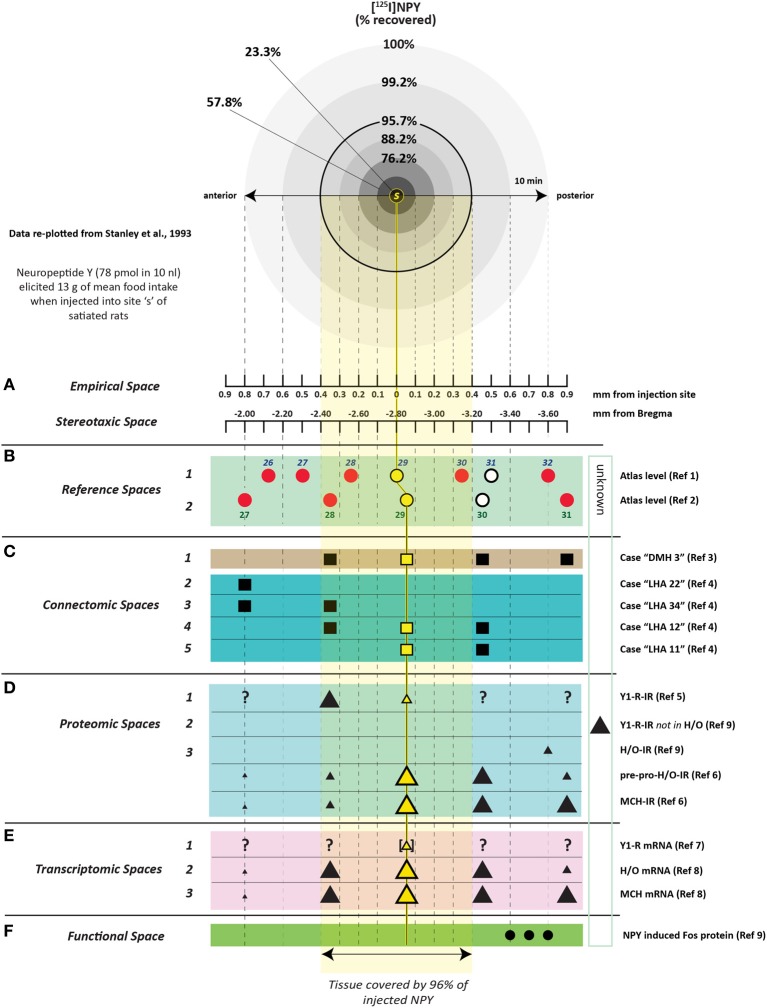
**An example of how visualizable spaces can be aligned to help identify neural substrates underlying feeding triggered by an injection of neuropeptide Y**. Data in the radial spread at the top of the figure are derived from the diffusion data of Stanley et al. (1993b) shown in Figure 3 in this article. The distance traveled by the diffusing solution of injected radiolabeled neuropeptide Y, is noted along a millimeter axis representing Empirical Space **(A)**. This space, in turn, is aligned with Stereotaxic Space (also panel **A**), and then aligned with **(B)** Reference Space 1 (Paxinos and Watson, 1986) and Reference Space 2 (Swanson, 2004). Alignment to these Reference Spaces allows further registration and alignment with datasets mapped to these spaces, including **(C)** Connectomic Spaces; **(D)** Proteomic Spaces; **(E)** Transcriptomic Spaces; and **(F)** Functional Space. Reference numbers listed on the right side of the figure refer to the following studies in this articles reference list: Ref 1: (Paxinos and Watson, 1986); Ref 2: (Swanson, 2004); Ref 3: Thompson et al. (1996); Ref 4: Hahn and Swanson (2010); Ref 5: Kopp et al. (2002); Ref 6: Hahn (2010); Ref 7: (Kishi et al., 2005); Ref 8: (Swanson et al., 2005); Ref 9: (Campbell et al., 2003). See text for a detailed explanation of this figure.

Given these facts, the schema in Figure 9 is presented. At the top of this figure, data obtained from the radiolabeling experiment (see Figure 3) of Stanley et al. (1993b) are re-plotted to reflect the hypothesis that the NPY is spreading from injection site “s,” which corresponds to the locus of NPY's effects as mapped in Figure 2H (see right side of Figure 2H; black circles representing intakes; the site that produced 13 g of food intake in 4 h). The percentages listed are those established by Stanley et al. (1993b), as the % of radiolabeled NPY recovered from the tissue (original inset **B** in Figure 3). The distance scale corresponding to the original data is re-plotted as the visualizable space called *empirical space*, in mm from the injection site. Given that the effective site is on a *PW* reference plate corresponding to a Bregma of −2.80 mm, this has been aligned to the *empirical space* as *stereotaxic space* (see panel **A** in Figure 9). In panel **B**, using the atlas conversion chart in Figure 8, *PW* atlas plates (labeled as “Reference Space 1”) are aligned with those *S* plates (“Reference Space 2”) that are in the vicinity of the rostrocaudal expanse traveled by the radiolabeled NPY.

#### 5.1.2. Alignment of additional visualizable spaces

The consequences of placing these levels from two *reference spaces* in register is apparent from the data summarized in panels C–F of Figure 9, which describe the locations of elements in *connectomic, proteomic, transcriptomic*, and *functional* visualizable spaces, respectively.

***5.1.2.1. Connectomic space alignment of traced circuits potentially affected by NPY injections***. In panel C, Thompson et al. (1996) used the anterograde tracer, PHA-L, to label cell bodies within the DMH, which they subsequently mapped to Level 29 of Swanson (case “DMH3” in their study). Similarly, Hahn and Swanson (2010) mapped PHA-L-labeled cells at Level 29, located within the lateral hypothalamic area; see cases “LHA11” and “LHA12” in panel B. The implications of placing these tracer data in register with the NPY injection site is clear: given the general anatomical proximity of the LHA and DMH to the injection site at Level 29, and the spread of the injected NPY, it is very likely that NPY solution spread to the areas where the anterogradely traced neurons are located. If NPY receptors are located on these neurons, then this would suggest further that these traced neurons could have been activated by the injected NPY. So the next question we can ask is: are NPY receptors located in the DMH and LHA, and if so, have they been mapped to Level 29?

***5.1.2.2. Chemoarchitectonic space alignments for NPY receptive neuronal phenotypes***. The Y1 subtype of the NPY receptor (Y1-R) is believed to mediate, in part, the feeding effects of NPY in rats and mice (Stanley et al., 1992; Lopez-Valpuesta et al., 1996; Kanatani et al., 2000; see Gehlert, 1999 for a discussion of the contributions of NPY receptor subtypes in food intake control). Knowing the distribution of NPY receptors, particularly the Y1-R, in relation to the location of the NPY injection site is important to begin understanding how NPY's orexigenic effects might be mediated. For *proteomic spaces* (panel **D**), recall from Section 4.6.2.1.1 that Kopp et al. (2002) reported the distribution of immunoreactivity for the Y1-R, and their inferred coordinates corresponded to Levels 28 and 29 of Swanson (2004) (e.g., see inferred Bregma coordinates listed at the upper right of Figure 7B). Thus, it is likely that Y1-Rs are expressed at these atlas levels.

Within *proteomic space*, however, it would be of further benefit if the neuronal phenotype (or phenotypes) that expresses the Y1-R has been identified at these atlas levels. What phenotypes are present at or near these levels and what is known about their abilities to respond to NPY via the Y1-R? Campbell et al. (2003) and Hahn (2010) have noted the existence of hypocretin/orexin (H/O)-expressing neurons in and around this region, and Hahn (2010) has also reported melanin concentrating hormone (MCH)-immunoreactive neurons at Levels 28 and 29. Along these same lines, panel **E** of Figure 9 shows “*transcriptomic spaces*” corresponding to the known distributions of mRNA for Y1-Rs (Kishi et al., 2005); H/O (Swanson et al., 2005); and MCH mRNAs (Swanson et al., 2005), which again corroborate distributions at these levels.

With respect to *proteomic space* and Y1-Rs on H/O or MCH phenotypes, a few other studies merit attention. Campbell et al. (2003) reported that Y1-R-immunoreactivity is not present on H/O neurons, but this observation was not specified to a rostrocaudal level. This absence of location data is noted as “unknown” (Figure 9, light blue box to the right). Fetissov et al. (2003) also reported the absence of Y1-Rs on H/O neurons. Y1-R presence or absence on H/O neurons notwithstanding, it also has been shown for mouse hypothalamic slices that the predominant electrophysiological action of NPY on H/O neurons is inhibitory (Fu et al., 2004). Assuming, therefore, that these findings generalize to rats and are also observed *in vivo*, any speculation on NPY triggering feeding through actions at H/O neurons would have to reconcile this direct *inhibitory* effect of NPY on H/O neurons with NPY's ability to *activate* a feeding circuit via the Y1-R following its central nanoinjection into the perifornical region. Indeed, Fu et al. (2004) also showed that Y1-R agonists reproduced NPY's inhibitory effect on H/O neurons: even if H/O neurons had Y1-Rs, they would likely be inhibited by these receptors. What about MCH neurons at these levels? Y1-Rs are also reportedly absent on MCH neurons [(Fetissov et al., 2003); unpublished observations of Campbell et al. (2003), p. 1495], and again, Fu et al. (2004) reported that NPY directly inhibits MCH neurons, so any model suggesting MCH neurons could represent the neuronal phenotype mediating NPY's orexigenic actions must also incorporate this as a constraint. Whether putative orexigenic actions of NPY on H/O or MCH neurons involves inhibition of an inhibitory circuit (disinhibition) remains to be seen.

Fetissov et al. (2003) also reported the presence of Y1-Rs on neurons that express nitric oxide synthase (NOS). These neurons were found in the ventromedial hypothalamus, and to a lesser extent the perifornical hypothalamus, although it again is unclear at what rostrocaudal level within the hypothalamus these neurons are found. However, in their *Chemoarchitectonic Atlas of the Rat Brain*, Paxinos et al. (2009) provide photomicrographic documentation for neurons stained for NOS at an *inferred stereotaxic coordinate* of 2.913 mm posterior to Bregma (see their Figure 193). These neurons, which were stained for NOS using a nicotinamide adenine dinucleotide phosphate diaphorase (NADPH diaphorase) stain (which marks NOS in a manner that does not require antibody staining), were located within the ventromedial and perifornical hypothalamus, confirming the findings of Fetissov et al. Importantly, the *inferred stereotaxic space* helps to strengthen the likelihood that the Y1-R-positive NOS neurons identified by Fetissov et al. (2003) are at or near Levels 28 or 29 of Swanson reference space.

*Functional space* has also been mapped in relation to NPY stimulation, which, according to the report from Campbell et al. (2003), triggered Fos protein at one level of *Reference Space 1* and two additional locations not registerable to any reference space level (Figure 9F). However, the investigators noted that intra-hypothalamic injection of NPY in the vicinity of the perifornical region did not trigger Fos expression in H/O or MCH neurons, consistent with the observations of Fu et al. (2004) that NPY inhibits these neurons. Finally, notice that the registration of all of these visualizable spaces allows us to extrapolate on the basis of Stanley et al. (1993b) that 96% of the radiolabeled NPY injected into site “s” will spread to rostral and caudal sites up to 0.4 mm away (as illustrated by the yellow box in Figure 9). Level 29 of Swanson marks the “epicenter” of the manipulation, and all cellular elements shown in yellow are located at the same anteroposterior level as the NPY injection site.

### 5.2. Neuroinformatics tools to automate the reasoning process

The example just worked through (Figure 9) illustrates the power of utilizing visualizable spaces, atlas registration, and empirical data concerning central injection of an orexigenic agent (e.g., NPY) to create a model where the influence of this neuropeptide is constrained across space and time to a few mapped cellular elements, some of which have established, traced connections to other regions of the brain. Ideally, then, such an exercise ought not to be performed by a human experimentalist but by a computational program that interacts with neuroinformatics-based databases containing records of curated experimental data from tract-tracing experiments.

The Swanson laboratory has constructed such a system for use with multiple animal models, especially the rat (Bota et al., 2012). Specifically, the Brain Architecture Management System (or BAMS; http://brancusi.usc.edu/), is a neuroinformatics workbench that allows one to query connections that have been mapped to the Swanson reference space or other reference spaces, including Paxinos and Watson reference spaces. As of 2013, over 70,000 connection reports exist in the BAMS database (Bota et al., 2012; Bota and Talpalaru, 2013). The main argument, then, for creating a system whereby information can be placed in basic registration with the Swanson reference space is to thereby access all the mapped connections in *S* space that are found in BAMS. Since multiple experimental datasets are present within BAMS, generated by diverse investigators, the mapping of one's experimental data to the *S* reference space allows one to participate in a community-based project focused on understanding brain circuits. A prototype co-registration system, called *NeuARt II* has been released by the Swanson group as a proof-of-concept that allows for the superimposition of location data from a variety of visualizable spaces (connectomic, proteomic, empirical, etc.) onto horizontal and transverse templates of *S* reference space (Burns et al., 2006). Other proofs-of-concept related to these efforts include electronic laboratory notebook tools and knowledge management systems to help sort and organize the data obtained for multiple visualizable spaces (Burns et al., 2003; Khan et al., 2006), and proposed formalizations of modeling language to manage connectivity data (Brown and Swanson, 2013). Finally, the second and third editions of the Swanson atlas (Swanson, 1999, 2004) include digital templates of each atlas plate in Adobe Illustrator format (discussed in Swanson, 2001). One can readily superimpose datasets onto these atlas plates by using the layer manager palette in Illustrator to overlay a transparent layer onto the atlas map and place location data in register with other data drawn onto different layers.

## 6. Concluding remarks

Pioneering creatures, when venturing or peering into uncharted places, usually return helpful information about the locations of such places to their companions. Subsequent journeys or observations then refine the initial maps that are formed of these places, in a form of culture- and community-based quorum sensing (Huth, 2013). Evidence for this is well documented for honeybees (von Frisch, 1953; Seeley and Visscher, 2004; Visscher, 2007; Seeley et al., 2012), cosmologists (Ferris, 1983; Rowan-Robinson, 1985), Antarctic explorers (Shackleton, 1919; Byrd, 1938; Sullivan, 1957; Pulsifer and Taylor, 2005; Pulsifer et al., 2005, 2007), and neuroscientists (Table 4; see also Figure 2 in Swanson, 2000b). Members of the last group, venturing out for their first time, are just as likely as the first three groups, if not more, to become lost if the valuable spatial information provided by their pioneers is ignored, forgotten, or misunderstood.

## 7. Note added in proof

The Stuber laboratory has recently reported that optogenetic stimulation of GABAergic neurons in the bed nucleus of the stria terminalis suppresses the activity of lateral hypothalamic glutamatergic neurons to stimulate food intake (Jennings et al., 2013). In their published Supplementary Material, optical fiber placements are plotted in gross anatomic and stereotaxic space.

### Conflict of interest statement

The author declares that the research was conducted in the absence of any commercial or financial relationships that could be construed as a potential conflict of interest.
